# Synthesis, in vitro α-glucosidase inhibitory activities, and molecular dynamic simulations of novel 4-hydroxyquinolinone-hydrazones as potential antidiabetic agents

**DOI:** 10.1038/s41598-023-32889-7

**Published:** 2023-04-18

**Authors:** Nahal Shayegan, Sirous Haghipour, Nader Tanideh, Ali Moazzam, Somayeh Mojtabavi, Mohammad Ali Faramarzi, Cambyz Irajie, Sara Parizad, Shirin Ansari, Bagher Larijani, Samanehsadat Hosseini, Aida Iraji, Mohammad Mahdavi

**Affiliations:** 1 Endocrinology and Metabolism Research Center, Endocrinology and Metabolism Clinical Sciences Institute, Tehran University of Medical Sciences, Tehran, Iran; 2 Stem Cells Technology Research Center, Shiraz University of Medical Sciences, Shiraz, Iran; 3 Department of Pharmaceutical Biotechnology, Faculty of Pharmacy, Tehran University of Medical Sciences, Tehran, Iran; 4 Department of Medical Biotechnology, School of Advanced Medical Sciences and Technologies, Shiraz University of Medical Sciences, Shiraz, Iran; 5 Student Research Committee, Shiraz University of Medical Sciences, Shiraz, Iran; 6 Central Research Laboratory, Shiraz University of Medical Sciences, Shiraz, Iran

**Keywords:** Chemical biology, Enzymes

## Abstract

In the present study, new structural variants of 4-hydroxyquinolinone-hydrazones were designed and synthesized. The structure elucidation of the synthetic derivatives **6a–o** was carried out using different spectroscopic techniques including FTIR, ^1^H-NMR, ^13^C-NMR, and elemental analysis, and their α-glucosidase inhibitory activity was also determined. The synthetic molecules **6a–o** exhibited good α-glucosidase inhibition with IC_50_ values ranging between 93.5 ± 0.6 to 575.6 ± 0.4 µM as compared to the standard acarbose (IC_50_ = 752.0 ± 2.0 µM). Structure–activity relationships of this series were established which is mainly based on the position and nature of the substituent on the benzylidene ring. A kinetic study of the active compounds **6l** and **6m** as the most potent derivatives were also carried out to confirm the mode of inhibition. The binding interactions of the most active compounds within the active site of the enzyme were determined by molecular docking and molecular dynamic simulations.

## Introduction

Diabetes mellitus (DM) is one of the main causes of mortality worldwide. According to Pan American Health Organization (PAHO), in 2019, the age-standardized death rate due to DM was estimated at 20.9 deaths per 100,000 population and it will keep rising over the next decades. DM categorize as a metabolic disorder corresponding with prolonged high blood sugar levels^1^. Hyperglycemia is the most critical criterion of all DM types and its consistency leads to various complications such as cardiovascular disorders, kidney failure, neuropathy, lipid metabolism disorders, and etc.^2,3^. Depending on the mechanism by which it occurs, DM is classified as Type I DM (T1DM), Type II DM (T2DM), and gestational DM (GDM) in which T2DM accounts for around 90% of DM cases^4^.

T2DM is characterized by insulin resistance in the target organs, declining insulin production, and eventual pancreatic β-cell failure^5^. Given inadequate levels of insulin and increased insulin resistance, results in hyperglycemia. In this context, enzymes involved in the regulation of postprandial hyperglycemia have been recognized as the candidate to target T2DM. α-glucosidase is a membrane-bound enzyme, localized in the epithelium of the small intestine that digests oligosaccharides and disaccharides into simple glucose, after which it gets absorbed and enters the bloodstream^6^. Inhibition of the α-glucosidase enzyme can help in delaying the digestion of carbohydrates, thereby reducing the levels of glucose in the blood. As a result, α-glucosidase inhibitors are of great interest as an efficacious and safe therapy for T2DM^7^. Acarbose, miglitol, and voglibose are approved drugs used as α-glucosidase inhibitors to target T2DM; unfortunately, these compounds are associated with deleterious side effects such as abdominal distention, bloating, and diarrhea^8,9^. Thus, the discovery of new, abundant, and safe α-glucosidase inhibitors is highly needed^10^.

**Figure 1:**
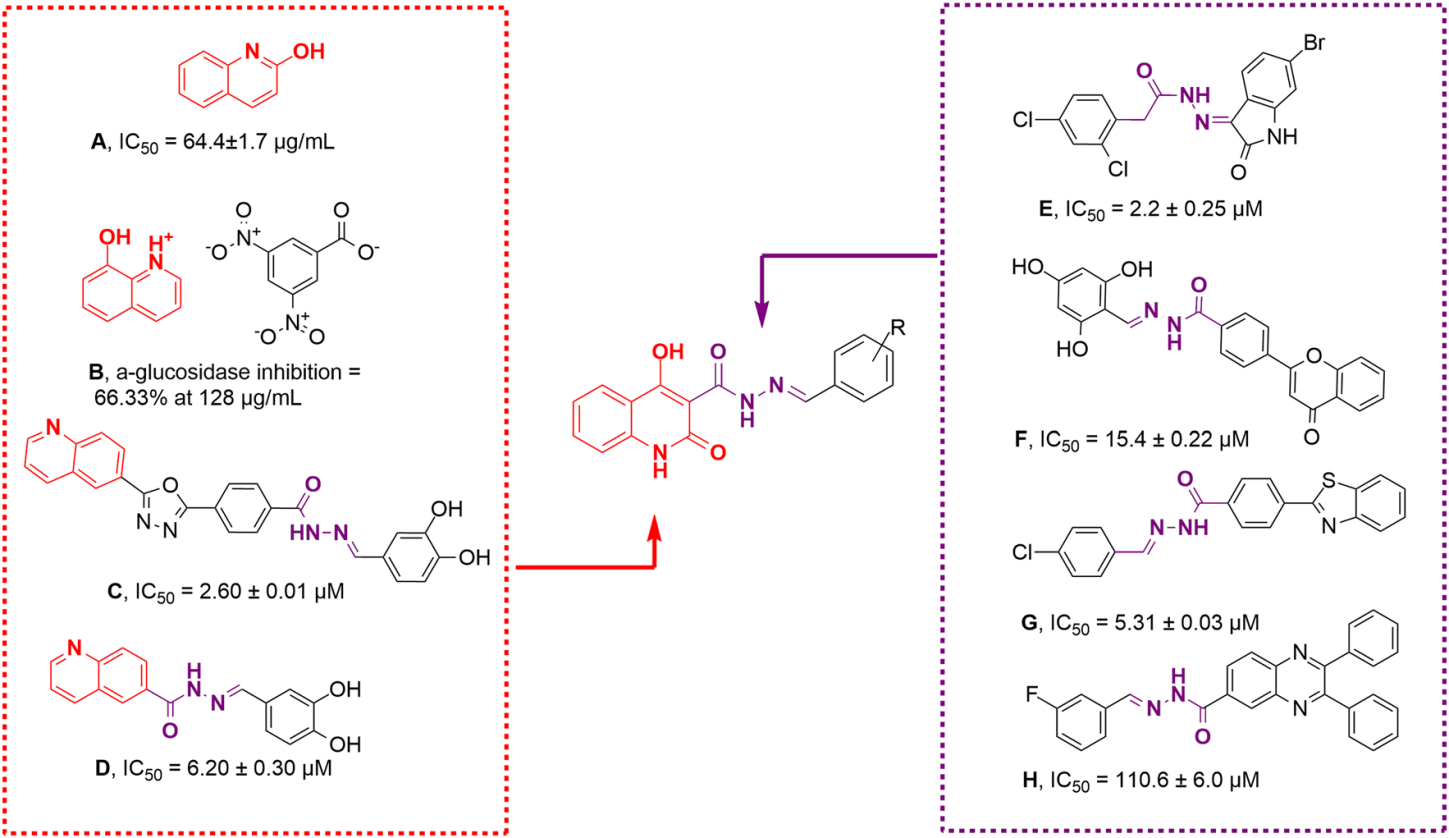
The rationale for the design of 4-hydroxyquinolinone hydrazone hybrids as new α-glucosidase inhibitors.

Nitrogen-containing compounds such as hydroxyquinolinone scaffolds are important structures found in many pharmaceutically active compounds with drug-like properties, which provide high-quality leads and compound libraries^11,12^. They are structurally diverse and possess a variety of biological activities such as anticancer, antimicrobial^13^, metal chelator^14^, antialzheimer^15^, and antifungal activities^16,17^. Recently, researchers had focused on the antidiabetic bioactivities of hydroxyquinolinone. In 2015, it was a report on α-amylase and α-glucosidase inhibitory activities of 2-hydroxyquinoline and its structural analogs. structure–activity relationships (SARs) revealed that 2-hydroxyquinoline (compound **A**, Fig. 1) analogs had potent inhibitory activity, while 2-methyl-8-hydroxyquinoline showed weak inhibition which demonstrated the critical role of OH moiety of the quinolone ring^18^. Satheesh et al., reported anti-inflammatory and α-glucosidase inhibitory activities of organic salt, 8-hydroxyquinolinium 3,5-dinitrobenzoate (compound **B**, Fig. 1). This compound exhibited 53.63% α-glucosidase inhibition at 128 μg/mL vs acarbose with 75.55% inhibition at the same concentration^19^. Quinoline derivatives were also reported as potent α-glucosidase inhibitors. In 2015, Taha et al. firstly, reported quinoline–oxadiazole Schiff base derivatives (compound **C**, Fig. 1) as potent α-glucosidase inhibitors with IC_50_ in the ranges between 2.60 to 102.12 μM in comparison with standard inhibitors with IC_50_ value of 38.25 ± 0.12 μM^20^. Compound **D** also demonstrated a good anti-α-glucosidase profile with IC_50_ of 6.20 to 48.20 µM compared to acarbose (IC_50_ = 38.45 µM). In silico study showed that the nitrogen and double bonded oxygen atom of quinoline rings of the compound form hydrogen bond with active site residues which might be one of the reasons showing the highest activity in the series^21^.

There are a bunch of reports in the literature, in which some hydrazone-containing compounds have shown potency as α-glucosidase inhibitors. Take the example of the recent research, compound **E** with isatin-hydrazone structure showed potent α-glucosidase inhibitory potential with IC_50_ value ranging between 2.2 ± 0.25 to 83.5 ± 1.0 µM. Also, flavone-hydrazone (compound **F**, Fig. 1) is categorized as another potent α-glucosidase inhibitor^22^. Series of benzothiazole-bearing benzohydrazide (compound **G**, Fig. 1) as α-glucosidase inhibitors showed good potency with IC_50_ values ranging between 5.31 and 53.34 μM^23^. Compound **H**, as a potent inhibitor with the competitive mode of inhibition, exhibited an IC_50_ value of 110.6 µM in comparison with acarbose (IC_50_ = 750.0 µM)^24^. Also, the potency of hydrazone linker was exhibited by participating in several interactions with the binding site of the enzyme^25^.

Although it was proposed that quinolines can reduce hyperglycemia via inhibiting α-glucosidase; there are limited data about the α-glucosidase potencies of hydroxyquinolinone with no report on 4-hydroxyquinolin-2(1*H*)-one as α-glucosidase inhibitor. As a result in a search for a new lead candidate, a novel series of 4-hydroxyquinolin-2(1*H*)-one bearing aryl hydrazone moieties were designed. Considering that the literature review has shown that the introduction/modification of specific groups on aromatic moiety may affect their biological activities, a series of 4-hydroxyquinolinone hydrazone hybrids were synthesized and evaluated for their α-glucosidase inhibition to establish the SARs. Also, the most potent derivatives were subjected to molecular docking and molecular dynamics simulations as well as kinetic studies to determine their behavior within the active site of the enzyme and type of inhibition.

## Results and discussion

### Chemistry

**Scheme 1:**
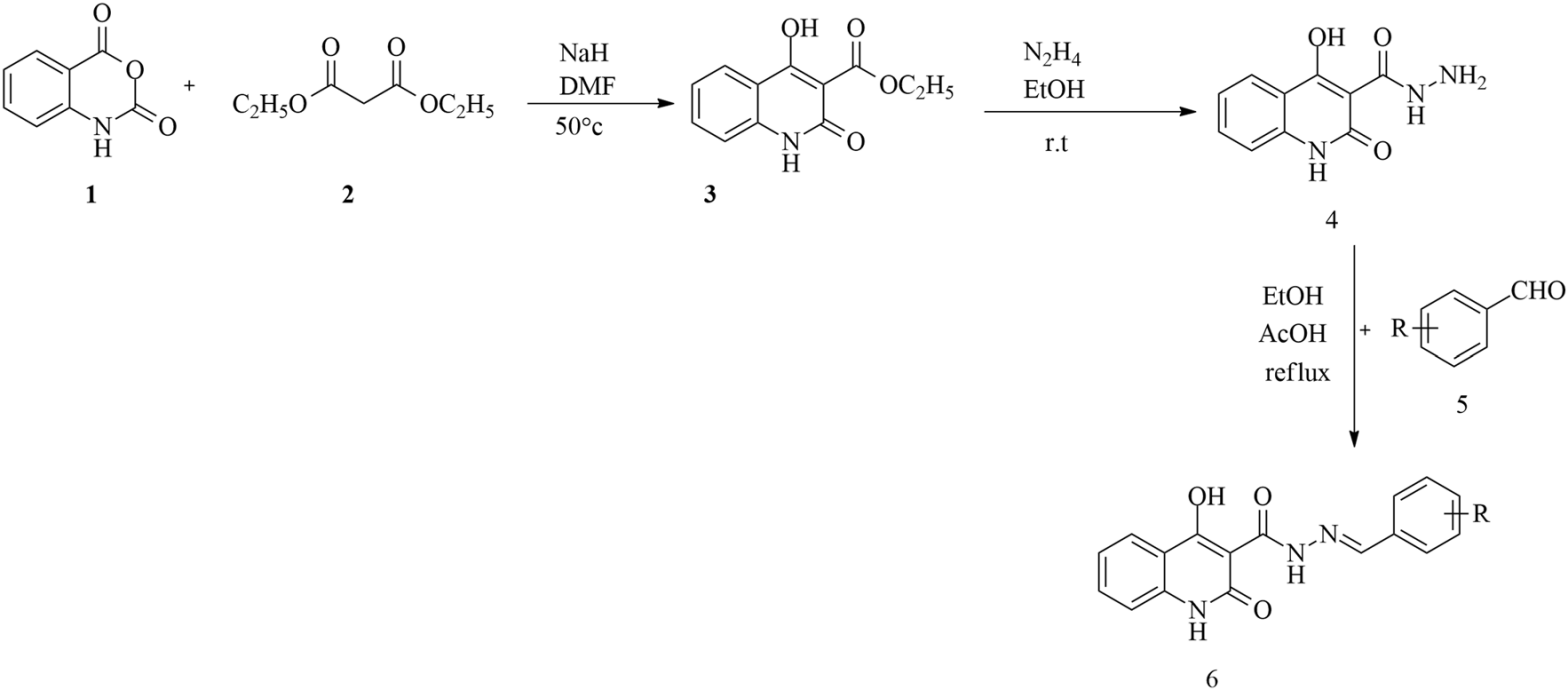
Synthesis of compounds **6a–o**.

Synthesis of the title compounds **6a–o** was schematically described in Scheme 1. It was initiated by the reaction of isatoic anhydride (**1**), diethyl malonate (**2**), and NaH in DMF at 50˚ leading to the formation of ethyl 4-hydroxy-2-oxo-1,2-dihydroquinoline-3-carboxylate **3**. Compound **4** in turn was prepared from the reaction of compound **3** and hydrazine hydrate in EtOH at room temperature. Finally, compound **4** was reacted with different aromatic aldehydes **5a–o** in ethanol, heated at reflux catalyzed by acetic acid to afford the corresponding Schiff base final products. All synthesized compounds were characterized by FTIR, ^1^H-NMR, ^13^C-NMR, and elemental analysis.

In the first step, the mechanism of the reaction involves trapping the proton from the C–H acid compound using NaH, followed by the nucleophilic attack to the isotopic anhydride, opening the ring and consequently removing OEt and CO_2_ groups to form compound **3**. In the second step, the nucleophilic attack of hydrazine hydrate to the carbonyl of the ester group led to the synthesis of compound **4**. In the last step, condensation of hydrazone with aldehydes under reflux conditions produce compound **6**. NMR results confirmed the structures of all synthesized compounds, for example in ^1^H NMR individual peaks such as the OH group appears in 13–14 ppm, the NH amide group appears in 12–13 ppm, another NH amide group appears in 8–9 ppm and imine group appears in aromatic region. Also, in ^13^C NMR individual peaks such as carbonyl of amide groups appear in 160–170 ppm, carbon of 4-hydroxyquinolinone appears in 170–175 ppm, and carbon of imine group appears in 145–150 ppm.

### In-vitro α-glucosidase inhibition

In continuation of our work to design α-glucosidase inhibitors, compounds **6a–o** were designed and synthesized. The result in Table 1 showed all compounds synthesized in this study are more active than the standard drug, acarbose. Concentrations of precipitation of all derivatives were more than 600 µM.

**Table 1:** α-Glucosidase inhibition of 4-hydroxyquinolinone hydrazone hybrids^a^.


Compounds	R	IC_50_ (µM)
6a	H	382.6 ± 0.9
6b	2-F	247.0 ± 1.1
6c	4-F	195.3 ± 1.2
6d	4-Cl	131.6 ± 0.3
6e	3-Br	436.7 ± 1.0
6f	4-Br	520.2 ± 1.5
6g	4-NO_2_	120.5 ± 0.8
6h	4-CH_3_	136.7 ± 0.5
6i	4-OCH_3_	259.0 ± 1.0
6j	2-OH	154.8 ± 0.1
6k	3-OH	196.3 ± 0.7
6l	2-OH-5-Br	94.2 ± 1.0
6m	2-OH-5-NO_2_	93.5 ± 0.6
6n	2,4-OH	187.8 ± 0.3
6o	3-OCH_3_-4-OH	575.6 ± 0.4
Acarbose		752.0 ± 2.0

^a^Data represented in terms of mean ± SD.

Results exhibited that one electron-withdrawing group had IC_50_ value in the range of 120.5 ± 0.8 to 520.2 ± 1.5 while derivatives with one electron donating group recorded IC_50_ values of 136.7 ± 0.5 to 259.0 ± 1.0 µM. Multi-substitution demonstrated various degrees of potency in the range of 93.5 ± 0.6 to 575.6 ± 0.4 µM.

It was observed that small electron withdrawing analogs such as 2-F (**6b**; IC_50_ = 247.0 µM), 4-F (**6c**; IC_50_ = 195.3 µM) and, 4-Cl (**6d**; IC_50_ = 131.6 µM) showed improved activity when compared to the unsubstituted derivative (**6a**, IC_50_ = 382.6 µM). However bulkier and more lipophilic electron-withdrawing groups reduced the potencies compared to unsubstituted derivatives. This trend can be seen in **6e** (R = 3-Br; IC_50_ = 436.7 µM) and **6f** (R = 4-Br; IC_50_ = 520.2 µM). NO_2_ substitution (**6g**; IC_50_ = 120.5 µM) resulted in the most potent derivatives among all electron-withdrawing groups and the third most active agents among all analogs.

Evaluation of analogs containing electron-donating substitutions showed that **6h** (R = 4-CH_3_; IC_50_ = 136.7 µM), **6j** (R = 2-OH; IC_50_ = 154.8 µM), and **6k** (R = 3-OH; IC_50_ = 196.3 µM) were highly potent against α-glucosidase; yet, derivative **6i** (IC_50_ = 259.0 µM) having 4-OCH_3_-group at *para*-position was found less inhibitory potential than other electron-donating analogs. These derivatives still demonstrated several-fold improvement in potency compared to the positive control.

Next, the multi-substitutions on the benzylidene ring were also evaluated. It was observed that analogs with 2 and 5 substituted groups on the aromatic ring displayed auspicious inhibitory activity and striking similarities in the inhibitory potential of 2-OH-5-Br (**6l**) and 2-OH-5-NO_2_ (**6m**) substituted analogs. Also, **6n** derivative (IC_50_ = 187.8 ± 0.3 µM) with 2,4-OH moiety raised the potency compared to the unsubstantiated one. However, **6o** analog with 3-OCH_3_-4-OH groups on the benzylidene ring displayed undesirable inhibitory activity.

**Figure 2:**
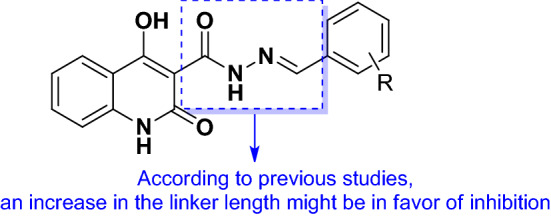
SARs studies of the current study and previously reported compounds.

Comparison of derivatives exhibited in Fig. 1 and our active compounds showed that **6l** and **6m** were more potent than entries **A**, **B**, and **D** compared with their related positive controls. However, these derivatives were not successful to improve inhibitory activities vs **C**, **E**, **F** and **G**. Although the backbones of these derivatives are not similar to fully extract SARs; however, it seems that the increase of the linker between the benzylidene ring (substituted moiety) and 4-hydroxyquinoline (aromatic ring) improves the potency which might be due to the better occupation of the binding site (Fig. 2).

### Enzyme kinetic studies

**Figure 3:**
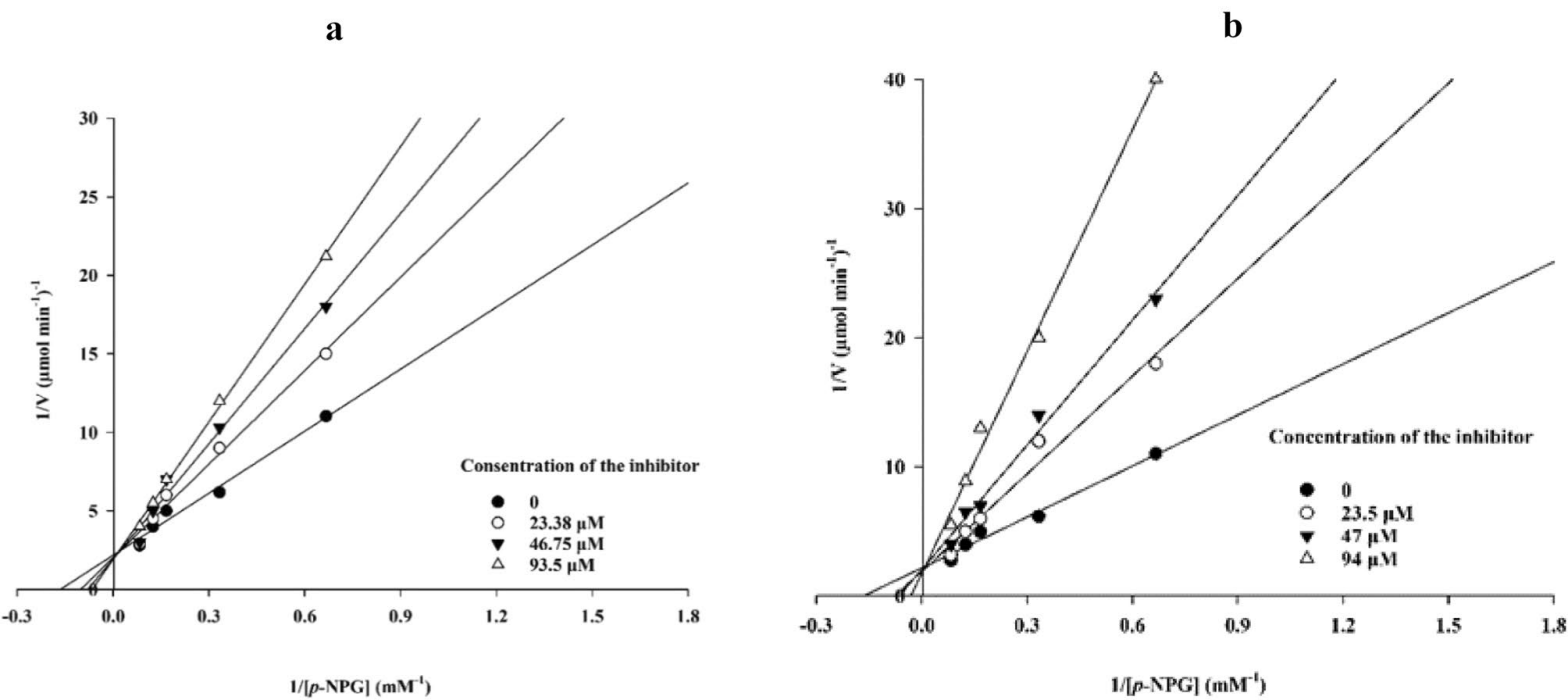
Kinetics of α-glucosidase inhibition by inhibitor. (**a**) The Lineweaver–Burk plot in the absence and presence of different concentrations of the **6m** (**b**) The Lineweaver–Burk plot in the absence and presence of different concentrations of the **6l**.

**Figure 4:**
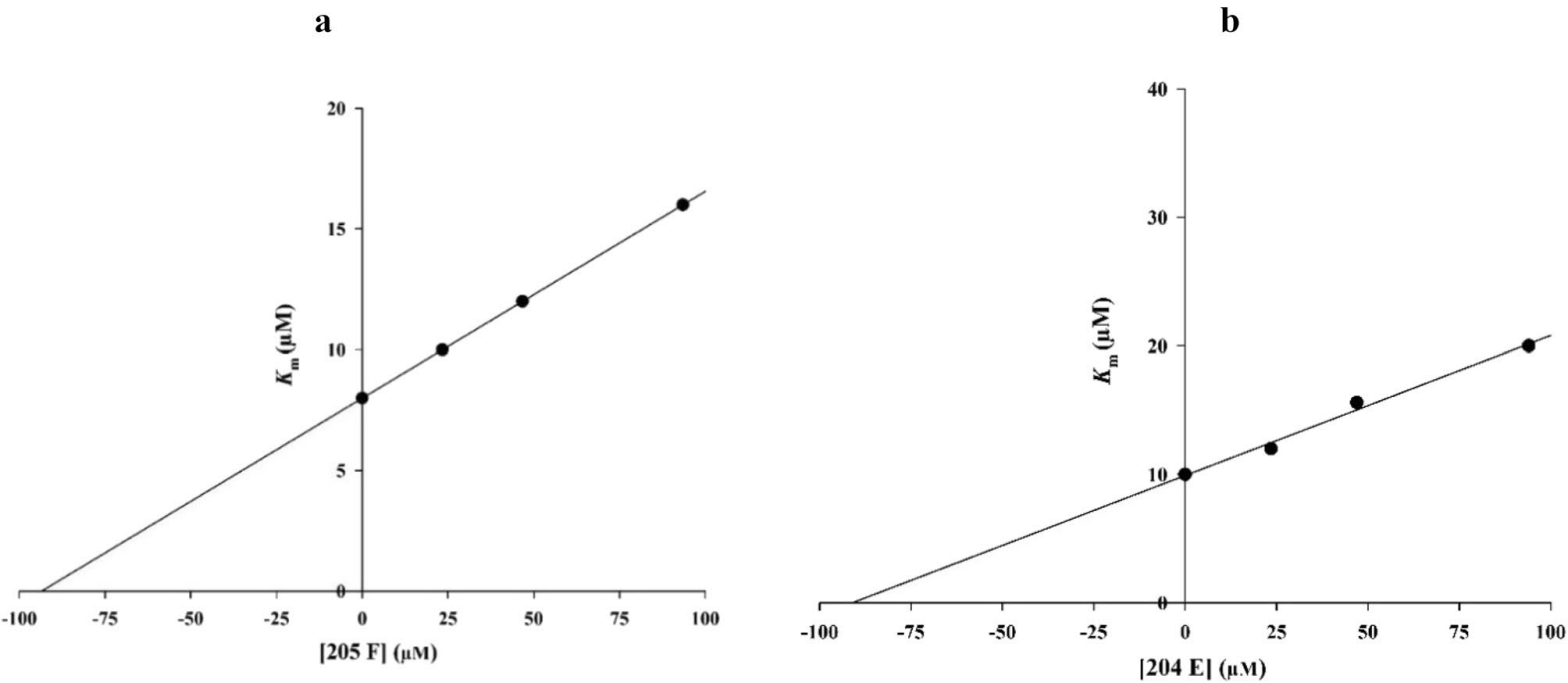
(**a**) The secondary plot between *K*_*m*_ and various concentrations of **6m**; (**b**) The secondary plot between *K*_*m*_ and various concentrations of **6l**.

The kinetic studies of **6l** and **6m** as the most potent derivative were executed. According to, the Lineweaver–Burk plot of **6m** (Fig. 3a) and **6l** (Fig. 3a), *K*_m_ gradually increased and *V*_*max*_ remained unchanged with increasing inhibitor concentration indicating a competitive inhibition. The results showed that **6m** and **6l** bind to the active site on the enzyme and compete with the substrate for binding to the active site. Furthermore, the plot of the *K*_m_ versus different concentrations of **6m** gave an estimate of the inhibition constant, *K*_i_ of 93.0 µM (Fig. 4a) and **6l** recorded the inhibition constant value of 92.0 µM (Fig. 4b).

### Molecular docking

Regarding that the X-ray crystallographic structure of α-glucosidase of *S. cerevisiae* being unavailable, the computational study was accomplished using the homology-modeled enzyme as reported in our articles^26^. It was shown that the α-glucosidase active site contains conserved residues around the substrate-binding site^27^ in which the active site pocket of the enzyme consists of a functional site lid at the interface of domain A and B and four subsides involve in substrate binding. The − 1 and + 1 subsides consist of Asp68, Tyr71, His111, Phe177, Gln181, Asp214, Glu276, Arg348 and Asp349. The + 2 subside comprise of Phe157, His239, Asn241 and Ala278, and the + 3 subside define by His279, Glu304, Thr307 and Phe311. Interactions with these regions might inhibit the activity of α-glucosidase. The results of the docking score of all derivatives are exhibited in Supplementary Table S1. **6l** and **6m** as the most potent derivatives recorded good values with glide scores of − 8.29 and − 8.14 kcal/mol and the least potent compounds **6o, 6e,** and **6f** demonstrated glid scores of − 5.22, − 5.26 and − 6.23 kcal/mol. Overall the same trend in the binding energy and inhibitory potency of derivatives was seen. The detailed interactions of **6l** and **6m** derivatives in the active site of the α-glucosidase enzyme are presented in Table 2.

**Table 2:** The predicted binding energy of **6l** and **6m** derivatives with the desired enzyme.

Compound	Moiety	Residue	Type of interaction	Distance (Å)
**6l**	Quinolinone	Phe310	Aliphatic interaction	3.45
	Quinolinone	Tyr313	Aliphatic interaction	3.32
	Quinolinone	Arg312	Salt bridge	4.33
	Quinolinone	Ash408	H-bound	2.53
	C=O	Ash408	H-bound	1.83
	OH	Asp349	H-bound	1.83
	OH	Ash439	H-bound	2.07
**6m**	Quinolinone	Hie239	Pi-cation	3.37
	Quinolinone	Lys155	H-bound	1.92
	OH	Ash408	H-bound	2.56
	Benzylidene	Ash408	Aliphatic interaction	3.15
	NO_2_	Asp349	Salt bridge	3.83
	NO_2_	Arg439	Salt bridge	2.43

**Figure 5:**
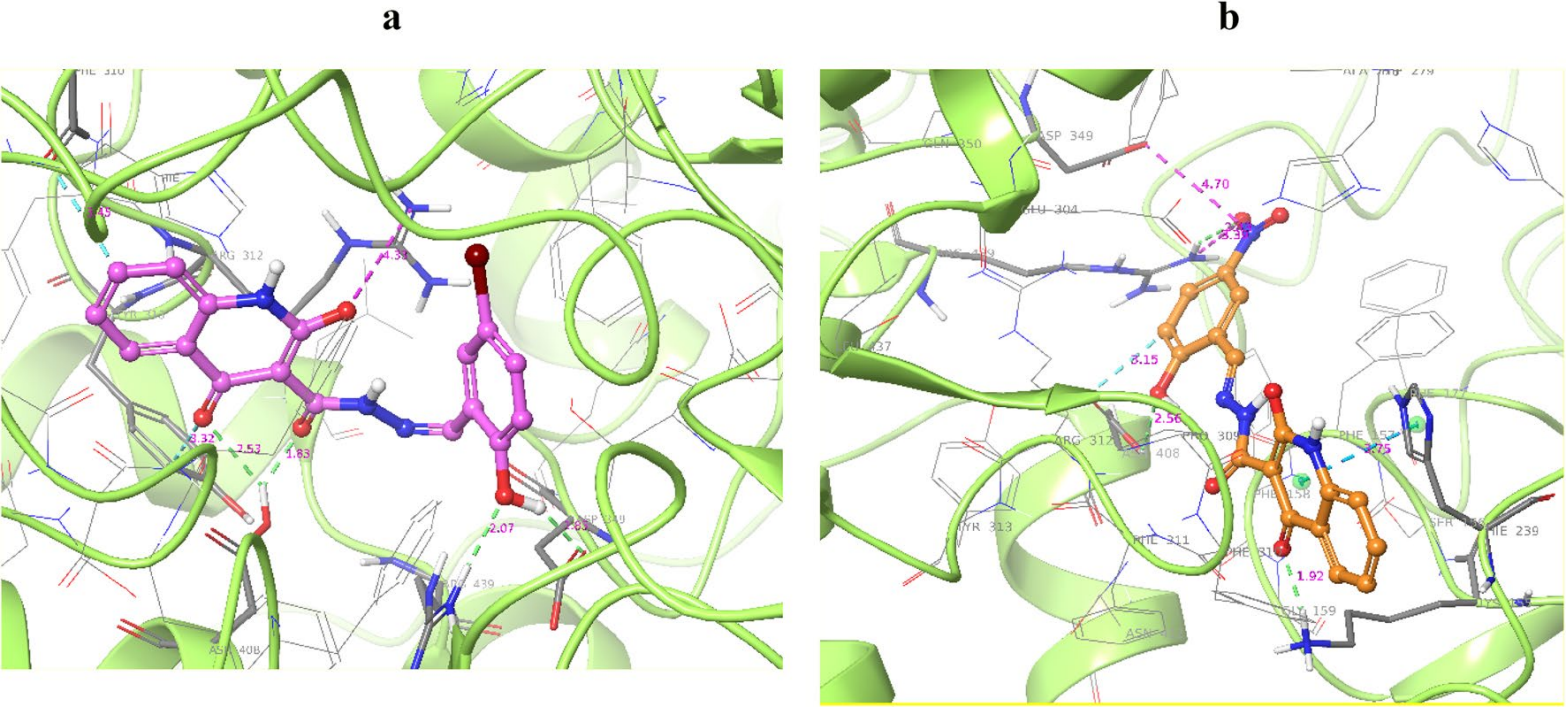
The pose and interaction pattern of **6l** (**a**) and **6m** (**b**) in the α-glucosidase enzyme.

As could be seen in Fig. 5a, 6l compound properly occupied the binding pocket of the enzyme and demonstrated four hydrogen bond interactions with the A subside. **6m** (Fig. 5b) placed well in the active site of the enzyme and benzylidene ring oriented toward the A domain subside whereas quinolinone participated in interaction with B domain side and loop which play a critical role in the hydrolysis process.

**Figure 6:**
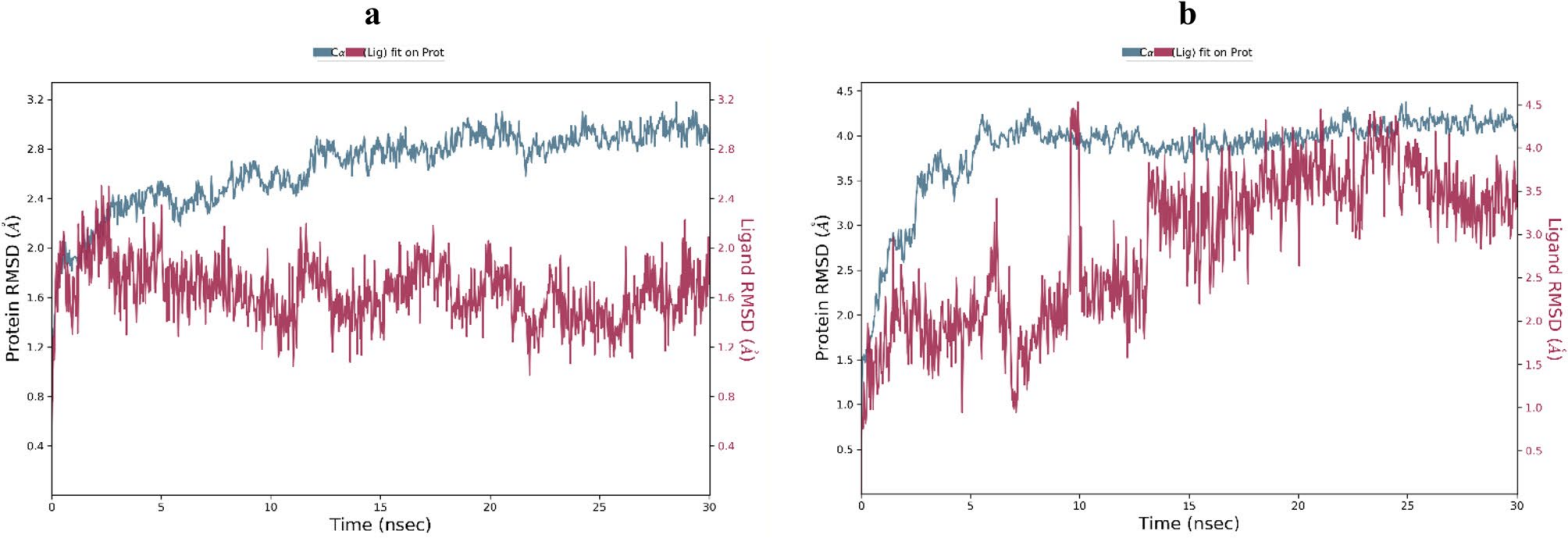
RMSD plot of the α-glucosidase backbone in complexed with **6l** (**a**) and **6m** (**b**).

IFD calculation was followed by mmgbsa/pbsa for the estimation of ∆G_binding_. ΔG_bind_ of α-glucosidase with compound **6l** complex and α-glucosidase with **6m** complex were estimated to be − 39.51 and − 31.37 kcal/mol, respectively. These results reveal stabilities and strong binding interactions of compounds **6l** and **6m** with the enzyme which is also supported by experimental assay.

### Molecular dynamic simulations

In order to investigate the binding interaction and the stability of the most potent compounds over the α-glucosidase active, molecular dynamics simulations were performed. The Root Mean Square Deviation (RMSD) of the protein’s backbone from its initial to final conformation was used to study the stability of the protein–ligand complex. The RMSD simulation showed α-glucosidase complexed with **6l** got overall stability after 2 ns of MD simulation time with RMSD stabilizing at an average of 1.6 Å (Fig. 6a). In contrast derivative **6m** reached stability after 15 ns with RMSD value of 3.2 Å (Fig. 6b). These results indicated that the α-glucosidase-**6l** complex was more stable during the simulation time compared with the α-glucosidase-**6m** complex.

**Figure 7:**
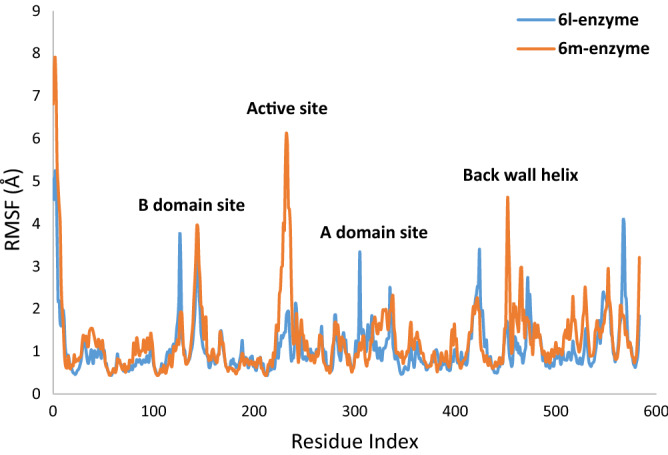
RMSF plot of the α-glucosidase residue in complexed with **6l** and **6m**.

The root mean square fluctuation (RMSF) value defines as the fluctuation of the protein’s residues from its average position throughout the simulation, which represents the flexibility of protein structure. Based on the timeline result, compound **6l** more effectively participated in interactions with the α-glucosidase binding site vs **6m** (Fig. 7). **6l** interacted with the residue of the active site and B domain site and reduce the fluctuations of the mentioned sites. Backing to Fig. 7, compound **6m** provides higher interaction with the B-domain site and A-domain site back-wall of the active.

**Figure 8:**
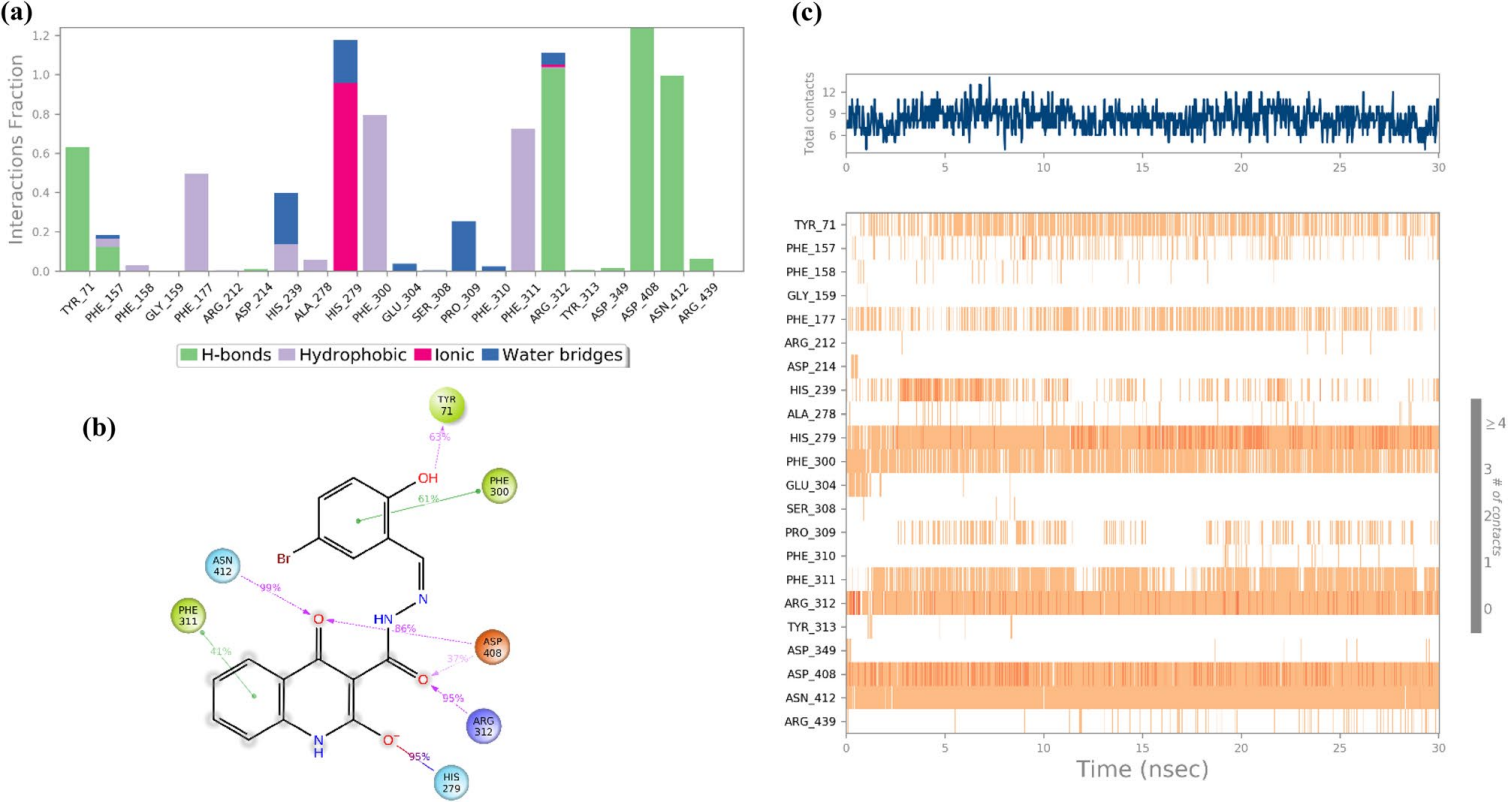
Type of interactions of compound **6l** within α-glucosidase (**a**), 2D representation of ligand-residue interactions that occur at least 40% of simulation time at the equilibrated phase of MD simulation which include α-glucosidase bound-state with compound **6l** (**b**) and timeline rendering of interacting residues during the whole simulation time (**c**).

Furthermore, Fig. 8 illustrates the timeline interactions of the quinolinone moiety of compound **6l** with His279 (92% of the simulation time), Asn412 (99% of the simulation time), and Phe311 (41% of the simulation time). On the other side of the molecule, 2-bromo-2-hydroxybenzylidene group disclosed interactions with Tyr71 (63% of the simulation time) and Phe300 (91% of the simulation time). The carbonyl linker group also recorded two H-bounds with Asp408 (37% of MD simulation time) and Arg312 (95% of MD simulation time).

**Figure 9:**
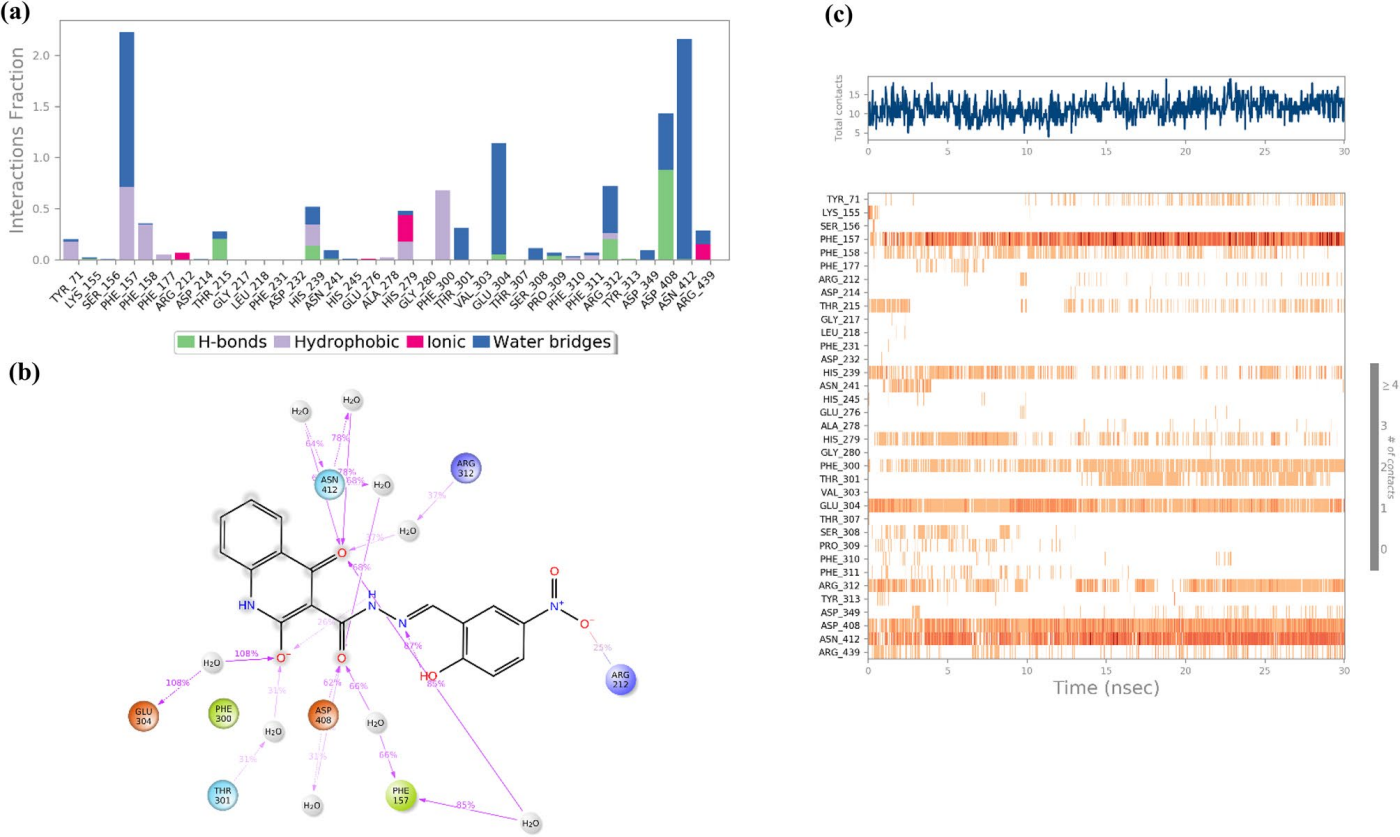
Type of interactions of compound **6m** within α-glucosidase (**a**), 2D representation of ligand-residue interactions that occur at least 25% of simulation time at the equilibrated phase of MD simulation which include α-glucosidase bound-state with compound **6m** (**b**) and timeline rendering of interacting residues during the whole simulation time (**c**).

As it is obvious in Fig. 9, compound **6m** coordinated in a way that the 2-bromo-5-nitrobenzylidene moiety exhibited interactions with Arg212 and hydrazide linker recorded interaction with Asp408 (62%) and Phe157 (66%) mediated with water. Quinazolinone aromatic rings provided two H-bound interactions with Asn412, Arg312, and Glu304 mediated with water.

## Conclusion

In conclusion, facile synthesis procedures were developed to synthesize 4-hydroxyquinolinone hydrazone derivatives. Different spectroscopic techniques such as FTIR, ^1^HNMR, ^13^CNMR, and elemental analysis were used to characterize the newly synthesized compounds. NMR results confirmed the structures of all synthesized compounds and ^1^H NMR and ^13^C NMR individual peaks such as OH/C–O group, amide groups, and imine group appear in their corresponding ppm. Next, all derivatives of 4-hydroxyquinoline-based hydrazone were evaluated in vitro α-glucosidase inhibition. All the newly afforded compounds were found to show moderate to good inhibitory activity against α-glucosidase when compared to standard acarbose. SARs showed that any type of substitution on the benzylidene ring improved the potencies and the best activities were observed in 2 and 5 di-substituted groups on the benzylidene ring. The kinetic studies of the most active compounds **6l** and **6m** showed that these compounds exhibit competitive modes of inhibitions. In addition, molecular docking studies were also conducted to explore the interactions of all derivatives against homology-modeled α-glucosidase, and the results supported the experimental data. MD study showed that the compound **6l** participated in several critical interactions with the residues of the binding site of enzymes including Tyr71, His279, Phe300, Phe311, Arg319 Asp408, and Asn412. Also, **6m** displayed prominent interactions with the α-glucosidase critical sites through hydrazone 2-hydroxy-5-nitrobenzylidene moiety involved in interaction with Arg212, Asp408, and Phe157 plus the 4-hydroxyquinolinone pharmacophore participated in three H-bound interactions with Asn412, Arg312, and Glu304.

Based on these results, 4-hydroxyquinolinone hydrazone derivatives could be considered a new candidate for further investigations as antidiabetic agents.

## Methods and materials

### General

All chemicals and reagents were purchased from Merck and Aldrich. The IR spectra were obtained on a Nicolet Magna FTIR 550 spectrometer (potassium bromide disks). Melting points were determined using Kofler hot stage apparatus and are uncorrected. NMR spectra were recorded on a Bruker 400 MHz.

### Synthesis

#### Synthesis of ethyl 4-hydroxy-2-oxo-1,2-dihydroquinoline-3-carboxylate (*3*)

A mixture of isatoic anhydride (**1**, 1 mmol, 0.163 g), diethyl malonate (**2**, 5 mmol, 0.76 mL), and NaH (2 mmol, 0.046 g) were added in DMF and the mixture was stirred at 50˚ for 12 h. The progress of the reaction was monitored by thin-layer chromatography (TLC). After completion of the reaction, H_2_O and then HCl was added until the precipitate was collected and filtration to give the desired compound in high yields. The precipitate was recrystallized using ethyl acetate.

#### Synthesis of 4-hydroxy-2-oxo-1,2-dihydroquinoline-3-carbohydrazide (*4*)

A mixture of ethyl 4-hydroxy-2-oxo-1,2-dihydroquinoline-3-carboxylate (**3,** 1 mmol), and hydrazine (1 mmol), in ethanol (5 ml) was stirred at room temperature for 24 h. The progress of the reaction was monitored by TLC. After completion of the reaction, water was added. The precipitate was collected, filtration and dried at room temperature. The precipitate was recrystallized using ethyl acetate.

#### Synthesis of *6a–o* derivatives

Different aldehydes (**5a–o**, 1 mmol) were added to the stirring solution of 4-hydroxy-2-oxo-1,2-dihydroquinoline-3-carbohydrazide (**4**, 1 mmol) and AcOH (10 drops) in ethanol (5 ml). The reaction was heated at reflux for 24 h. The progress of the reaction was monitored by TLC. After completion of the reaction, the mixture was poured over ice and water, and the precipitate was collected and filtered off to afford final products **6a–o**. The precipitate was recrystallized using ethyl acetate.

#### (*E*)-*N'*-benzylidene-4-hydroxy-2-oxo-1,2-dihydroquinoline-3-carbohydrazide (6a)

White solid. isolated yield: 74%, mp 175–177 °C. IR (KBr, υ): 3443, 3292, 1660, 1598, 1232 cm^−1^. ^1^H NMR (400 MHz, DMSO-*d*_6_) δ 13.32 (s, 1H, O–H), 12.08 (s, 1H, N–H), 8.49 (s, 1H, N–H), 8.01 (dd, *J* = 8.2, 1.4 Hz, 1H, C–H), 7.79 (dd, *J* = 7.7, 3.0 Hz, 2H, C–H), 7.73 (td, *J* = 7.6, 1.5 Hz, 1H, C–H), 7.49 (dd, *J* = 7.6, 1.9 Hz, 4H, C–H), 7.41 (d, *J* = 8.3 Hz, 1H, C–H), 7.36–7.29 (m, 1H, C–H). ^13^C NMR (100 MHz, DMSO-*d*_6_) δ 173.24, 167.94, 162.83, 151.34, 139.31, 134.75, 134.19, 131.15, 129.34, 128.03, 124.47, 123.14, 116.44, 114.56, 96.42 ppm. *Anal* Calcd. for C_17_H_13_N_3_O_3_, C, 66.44 H, 4.26, N, 13.67 found C, 66.70, H, 4.47, N, 13.85.

#### (*E*)-*N'*-(2-fluorobenzylidene)-4-hydroxy-2-oxo-1,2-dihydroquinoline-3-carbohydrazide (6b)

White solid. isolated yield: 87%, mp 190–192 °C. IR (KBr, υ): 3448, 3309, 1658, 1598, 1228, 976 cm^−1^. ^1^H NMR (400 MHz, DMSO-*d*_6_) δ 13.39 (s, 1H, O–H), 12.09 (s, 1H, N–H), 8.60 (s, 1H, N–H), 8.02 (d, *J* = 8.0 Hz, 1H, C–H), 7.99–7.87 (m, 1H, C–H), 7.74 (t, *J* = 7.7 Hz, 1H, C–H), 7.59–7.47 (m, 1H, C–H), 7.41 (d, *J* = 8.4 Hz, 1H, C–H), 7.33 (dd, *J* = 9.2, 5.7 Hz, 4H, C–H). ^13^C NMR (100 MHz, DMSO-*d*_6_) δ 172.21, 168.13, 163.70 (^1^*J*_C-F_ = 241 Hz), 162.33, 148.31, 140.30, 134.63 (^3^*J*_C-F_ = 9 Hz), 133.27 (^3^*J*_C-F_ = 9 Hz), 127.53, 125.48 (^4^*J*_C-F_ = 3 Hz), 124.51, 123.15, 119.40 (^2^*J*_C-F_ = 20 Hz), 116.75 (^2^*J*_C-F_ = 20 Hz), 116.46, 114.51, 95.01 ppm. *Anal* Calcd. for C_17_H_12_FN_3_O_3_, C, 62.77 H, 3.72, N, 12.92 found C, 62.57, H, 3.93, N, 13.11.

#### (*E*)-*N'*-(4-fluorobenzylidene)-4-hydroxy-2-oxo-1,2-dihydroquinoline-3-carbohydrazide (6c)

White solid. isolated yield: 85%, mp 192–194 °C. IR (KBr, υ): 3446, 3302, 1652, 1599, 1214, 973 cm^−1^. ^1^H NMR (400 MHz, DMSO-*d*_6_) δ 13.33 (s, 1H, O–H), 12.09 (s, 1H, N–H), 8.50 (s, 1H, N–H), 8.01 (dd, *J* = 8.1, 1.4 Hz, 1H, C–H), 7.92–7.75 (m, 2H, C–H), 7.73 (ddd, *J* = 8.4, 7.0, 1.5 Hz, 1H, C–H), 7.41 (d, *J* = 8.2 Hz, 1H, C–H), 7.37–7.27 (m, 4H, C–H). ^13^C NMR (100 MHz, DMSO-*d*_6_) δ 173.23, 167.93, 165.15 (^1^*J*_C-F_ = 248 Hz), 162.83, 150.23, 139.31, 134.75, 130.86 (^4^*J*_C-F_ = 3 Hz), 130.30 (^3^*J*_C-F_ = 8 Hz), 124.46, 123.13, 116.58 (^2^*J*_C-F_ = 22 Hz), 116.45, 114.56, 96.41 ppm. *Anal* Calcd. for C_17_H_12_FN_3_O_3_, C, 62.77 H, 3.72, N, 12.92 found C, 62.98, H, 3.59, N, 13.07.

#### (*E*)-*N'*-(4-chlorobenzylidene)-4-hydroxy-2-oxo-1,2-dihydroquinoline-3-carbohydrazide (6d)

White solid. isolated yield: 83%, mp 194–196 °C. IR (KBr, υ): 3449, 3308, 1658, 1597, 1207, 756 cm^−1^. ^1^H NMR (400 MHz, DMSO-*d*_6_) δ 13.36 (s, 1H, O–H), 12.10 (s, 1H, N–H), 8.51 (s, 1H, N–H), 8.02 (d, *J* = 8.4 Hz, 1H, C–H), 7.81 (d, *J* = 8.5 Hz, 2H, C–H), 7.74 (t, *J* = 6.9 Hz, 1H, C–H), 7.56 (d, *J* = 8.5 Hz, 2H, C–H), 7.41 (d, *J* = 8.3 Hz, 1H, C–H), 7.34 (t, *J* = 7.5 Hz, 1H, C–H). ^13^C NMR (100 MHz, DMSO-*d*_6_) δ 173.64, 167.25, 162.47, 150.65, 139.33, 136.34, 132.17, 130.90, 129.64, 128.25, 124.51, 123.21, 116.46, 114.55, 95.43 ppm. *Anal* Calcd. for C_17_H_12_ClN_3_O_3_, C, 59.75 H, 3.54, N, 12.30 found C, 59.87, H, 3.68, N, 12.14.

#### (*E*)-*N'*-(3-bromobenzylidene)-4-hydroxy-2-oxo-1,2-dihydroquinoline-3-carbohydrazide (6e)

White solid. isolated yield: 79%, mp 187–189 °C. IR (KBr, υ): 3439, 3293, 1647, 1603, 1226, 682 cm^−1^. ^1^H NMR (400 MHz, DMSO-*d*_6_) δ 13.39 (s, 1H, O–H), 12.11 (s, 1H, N–H), 8.48 (s, 1H, N–H), 8.01 (dd, *J* = 8.2, 1.3 Hz, 1H, C–H), 7.96 (t, *J* = 1.7 Hz, 1H, C–H), 7.80–7.66 (m, 4H, C–H), 7.46 (d, *J* = 7.8 Hz, 1H, C–H), 7.47–7.40 (m, 2H, C–H), 7.33 (t, *J* = 7.6 Hz, 1H, C–H). ^13^C NMR (100 MHz, DMSO-*d*_6_) δ 173.27, 168.10, 162.83, 149.65, 139.35, 136.66, 134.83, 133.61, 131.54, 130.17, 126.97, 124.49, 123.17, 122.60, 116.47, 114.51, 96.45 ppm. *Anal* Calcd. for C_17_H_12_BrN_3_O_3_, C, 52.87 H, 3.13, N, 10.88 found C, 52.65, H, 3.23, N, 11.01.

#### (*E*)-*N'*-(4-bromobenzylidene)-4-hydroxy-2-oxo-1,2-dihydroquinoline-3-carbohydrazide (6f)

White solid. isolated yield: 83%, mp 179–181 °C. IR (KBr, υ): 3451, 3307, 1661, 1601, 1202 cm^−1^. ^1^H NMR (400 MHz, DMSO-*d*_6_) δ 13.37 (s, 1H, O–H), 12.10 (s, 1H, N–H), 8.50 (s, 1H, N–H), 8.02 (d, *J* = 8.1 Hz, 1H, C–H), 7.72 (q, *J* = 8.7 Hz, 6H, C–H), 7.41 (d, *J* = 8.3 Hz, 1H, C–H), 7.33 (t, *J* = 7.6 Hz, 1H, C–H). ^13^C NMR (100 MHz, DMSO-*d*_6_) δ 173.27, 168.02, 162.83, 157.23, 150.21, 139.34, 134.78, 133.51, 132.40, 129.83, 124.49, 123.17, 116.46, 114.53, 96.44 ppm. *Anal* Calcd. for C_17_H_12_BrN_3_O_3_, C, 52.87 H, 3.13, N, 10.88 found C, 52.99, H, 3.01, N, 10.97.

#### (*E*)-4-hydroxy-*N'*-(4-nitrobenzylidene)-2-oxo-1,2-dihydroquinoline-3-carbohydrazide (6g)

Light brown solid. isolated yield: 89%, mp 208–210 °C. IR (KBr, υ): 3464, 3311, 1675, 1606, 1553, 1350, 1231 cm^−1^. ^1^H NMR (400 MHz, DMSO-*d*_6_) δ 13.52 (s, 1H, O–H), 12.14 (s, 1H, N–H), 8.65 (s, 1H, N–H), 8.34 (d, *J* = 8.3 Hz, 2H, C–H), 8.03 (d, *J* = 7.8 Hz, 3H, C–H), 7.81–7.69 (m, 1H, C–H), 7.49–7.06 (m, 3H, C–H). ^13^C NMR (100 MHz, DMSO-*d*_6_) δ 172.57, 167.30, 162.74, 154.81, 151.35, 139.89, 139.01, 134.94, 129.54, 127.83, 124.60, 123.29, 116.37, 114.66, 95.61 ppm. *Anal* Calcd. for C_17_H_12_N_4_O_5_, C, 57.96 H, 3.43, N, 15.90 found C, 58.13, H, 3.63, N, 16.06.

#### (*E*)-4-hydroxy-*N'*-(4-methylbenzylidene)-2-oxo-1,2-dihydroquinoline-3-carbohydrazide (6h)

White solid. isolated yield: 80%, mp 180–182 °C. IR (KBr, υ): 3455, 3297, 1666, 1600, 1247 cm^−1^. ^1^H NMR (400 MHz, DMSO-*d*_6_) δ 13.28 (s, 1H, O–H), 12.07 (s, 1H, N–H), 8.44 (s, 1H, N–H), 8.01 (dd, *J* = 8.2, 1.4 Hz, 1H, C–H), 7.80–7.61 (m, 3H, C–H), 7.41 (d, *J* = 8.3 Hz, 1H, C–H), 7.37–7.25 (m, 4H, C–H), 2.35 (s, 3H, CH_3_). ^13^C NMR (100 MHz, DMSO-*d*_6_) δ 173.22, 167.82, 162.83, 151.34, 141.11, 139.30, 134.71, 131.50, 129.94, 128.03, 124.46, 123.12, 116.45, 114.60, 96.41, 21.58 ppm. *Anal* Calcd. for C_18_H_15_N_3_O_3_, C, 67.28 H, 4.71, N, 13.08 found C, 67.45, H, 4.93, N, 13.26.

#### (*E*)-4-hydroxy-*N'*-(4-methoxybenzylidene)-2-oxo-1,2-dihydroquinoline-3-carbohydrazide (6i)

White solid. isolated yield: 78%, mp 189–191 °C. IR (KBr, υ): 3434, 3291, 1644, 1604, 1231, 1173 cm^−1^. ^1^H NMR (400 MHz, DMSO-*d*_6_) δ 13.24 (s, 1H, O–H), 12.07 (s, 1H, N–H), 8.42 (s, 1H, N–H), 8.01 (d, *J* = 8.0 Hz, 1H, C–H), 7.81–7.60 (m, 4H, C–H), 7.41 (d, *J* = 8.3 Hz, 1H, C–H), 7.33 (t, *J* = 7.6 Hz, 1H, C–H), 7.05 (d, *J* = 8.4 Hz, 2H, C–H), 3.82 (s, 3H, OCH_3_). ^13^C NMR (100 MHz, DMSO-*d*_6_) δ 173.19, 167.64, 162.84, 161.77, 151.17, 139.27, 134.67, 129.75, 126.72, 124.45, 123.11, 116.44, 114.86, 114.63, 96.39, 55.82 ppm. *Anal* Calcd. for C_18_H_15_N_3_O_4_, C, 64.09 H, 4.48, N, 12.46 found C, 63.86, H, 4.25, N, 12.28.

#### (*E*)-4-hydroxy-*N'*-(2-hydroxybenzylidene)-2-oxo-1,2-dihydroquinoline-3-carbohydrazide (6j)

White solid. isolated yield: 69%, mp 177–179 °C. IR (KBr, υ): 3440, 3291, 1641, 1601, 1232 cm^−1^. ^1^H NMR (400 MHz, DMSO-*d*_6_) δ 13.36 (s, 1H, O–H), 12.11 (s, 1H, N–H), 11.01 (s, 1H, O–H), 8.68 (s, 1H, N–H), 8.00 (d, *J* = 8.1 Hz, 1H, C–H), 7.72 (t, *J* = 7.6 Hz, 1H, C–H), 7.59 (dd, *J* = 7.7, 1.7 Hz, 1H, C–H), 7.49–7.26 (m, 4H, C–H), 6.95 (t, *J* = 7.7 Hz, 2H, C–H). ^13^C NMR (100 MHz, DMSO-*d*_6_) δ 173.03, 167.49, 162.75, 158.06, 151.22, 139.35, 134.76, 132.55, 130.08, 124.47, 123.12, 119.94, 118.87, 116.92, 116.46, 114.46, 96.39 ppm. *Anal* Calcd. for C_17_H_13_N_3_O_4_, C, 63.16, H, 4.05, N, 13.00 found C, 63.28, H, 4.19, N, 13.21.

#### (*E*)-4-hydroxy-*N'*-(3-hydroxybenzylidene)-2-oxo-1,2-dihydroquinoline-3-carbohydrazide (6k)

White solid. isolated yield: 72%, mp 186–188 °C. IR (KBr, υ): 3445, 3310, 1644, 1592, 1212 cm^−1^. ^1^H NMR (400 MHz, DMSO-*d*_6_) δ 13.28 (s, 1H, O–H), 12.06 (s, 1H, N–H), 9.71 (s, 1H, O–H), 8.39 (s, 1H, N–H), 8.00 (dd, *J* = 8.1, 1.4 Hz, 1H, C–H), 7.71 (ddd, *J* = 8.5, 7.1, 1.5 Hz, 1H, C–H), 7.40 (d, *J* = 8.3 Hz, 1H, C–H), 7.36–7.22 (m, 4H, C–H), 7.19 (dt, *J* = 7.7, 1.3 Hz, 1H, C–H), 6.87 (ddd, *J* = 8.0, 2.6, 1.1 Hz, 1H, C–H). ^13^C NMR (100 MHz, DMSO-*d*_6_) δ 173.23, 167.91, 162.81, 158.10, 151.38, 139.30, 135.46, 134.69, 130.37, 124.45, 123.08, 119.72, 118.48, 116.43, 114.57, 113.60, 96.41 ppm. *Anal* Calcd. for C_17_H_13_N_3_O_4_, C, 63.16, H, 4.05, N, 13.00 found C, 63.01, H, 3.87, N, 13.19.

#### (*E*)-*N'*-(5-bromo-2-hydroxybenzylidene)-4-hydroxy-2-oxo-1,2-dihydroquinoline-3-carbohydrazide (6l)

White solid. isolated yield: 80%, mp 171–173 °C. IR (KBr, υ): 3456, 3291, 1667, 1596, 1211, 646 cm^−1^. ^1^H NMR (400 MHz, DMSO-*d*_6_) δ 13.41 (s, 1H, O–H), 12.13 (s, 1H, N–H), 11.08 (s, 1H, O–H), 8.61 (s, 1H, N–H), 8.02 (d, *J* = 8.1 Hz, 1H, C–H), 7.88–7.60 (m, 2H, C–H), 7.56–7.18 (m, 4H, C–H), 6.92 (d, *J* = 8.8 Hz, 1H, C–H). ^13^C NMR (100 MHz, DMSO-*d*_6_) δ 173.13, 167.70, 162.77, 162.10, 157.07, 148.65, 139.37, 134.88, 134.74, 130.99, 124.52, 123.19, 121.36, 119.26, 116.46, 110.98, 96.44 ppm. *Anal* Calcd. for C_17_H_12_BrN_3_O_4_, C, 50.77 H, 3.01, N, 10.45 found C, 50.54, H, 3.16, N, 10.29.

#### (*E*)-4-hydroxy-*N'*-(2-hydroxy-5-nitrobenzylidene)-2-oxo-1,2-dihydroquinoline-3-carbohydrazide (6m)

Light brown solid. isolated yield: 81%, mp 198–200 °C. IR (KBr, υ): 3451, 3294, 1671, 1603, 1552, 1351, 1222 cm^–1^. ^1^H NMR (400 MHz, DMSO-*d*_6_) δ 13.48 (s, 1H, O–H), 12.14 (s, 1H, N–H), 8.70 (s, 1H, N–H), 8.56 (d, *J* = 2.9 Hz, 1H, C–H), 8.20 (dd, *J* = 9.1, 2.9 Hz, 1H, C–H), 8.02 (d, *J* = 8.1 Hz, 1H, C–H), 7.73 (ddd, *J* = 8.5, 7.0, 1.6 Hz, 1H, C–H), 7.42–7.31 (m, 3H, C–H), 7.12 (d, *J* = 9.1 Hz, 1H, C–H). ^13^C NMR (100 MHz, DMSO-*d*_6_) δ 173.08, 167.61, 162.18, 161.53, 154.70, 150.47, 139.44, 130.22, 127.67, 125.42, 124.31, 123.25, 120.02, 117.76, 116.43, 114.51, 95.20 ppm. *Anal* Calcd. for C_17_H_12_N_4_O_6_, C, 55.44 H, 3.28, N, 15.21 found C, 55.66, H, 3.12, N, 15.48.

#### (*E*)-*N'*-(2,4-dihydroxybenzylidene)-4-hydroxy-2-oxo-1,2-dihydroquinoline-3-carbohydrazide (6n)

White solid. isolated yield: 65%, mp 185–187 °C. IR (KBr, υ): 3467, 3288, 1637, 1605, 1243 cm^−1^. ^1^H NMR (400 MHz, DMSO-*d*_6_) δ 13.20 (s, 1H, O–H), 12.05 (s, 1H, N–H), 11.13 (s, 1H, O–H), 10.11 (s, 1H, O–H), 8.53 (s, 1H, N–H), 7.96 (d, *J* = 8.2 Hz, 1H, C–H), 7.68 (t, *J* = 7.7 Hz, 1H, C–H), 7.40–7.16 (m, 4H, C–H), 6.45–6.21 (m, 2H, C–H). ^13^C NMR (100 MHz, DMSO-*d*_6_) δ 172.87, 166.97, 162.73, 161.82, 160.12, 151.91, 139.24, 134.56, 132.03, 124.38, 123.02, 116.41, 114.51, 110.72, 108.45, 103.04, 96.31 ppm. *Anal* Calcd. for C_17_H_13_N_3_O_5_, C, 60.18 H, 3.86, N, 12.38 found C, 60.39, H, 4.00, N, 12.24.

#### (*E*)-4-hydroxy-*N'*-(4-hydroxy-3-methoxybenzylidene)-2-oxo-1,2-dihydroquinoline-3-carbohydrazide (6o)

White solid. isolated yield: 70%, mp 193–195 °C. IR (KBr, υ): 3436, 3297, 1651, 1597, 1192 cm^−1^. ^1^H NMR (400 MHz, DMSO-*d*_6_) δ 13.20 (s, 1H, O–H), 12.02 (s, 1H, N–H), 9.70 (s, 1H, O–H), 8.29 (s, 1H, N–H), 7.96 (d, *J* = 8.0 Hz, 1H, C–H), 7.68 (t, *J* = 7.7 Hz, 1H, C–H), 7.46–7.05 (m, 5H, C–H), 6.86 (d, *J* = 8.1 Hz, 1H, C–H), 3.83 (s, 3H, OMe). ^13^C NMR (100 MHz, DMSO-*d*_6_) δ 173.08, 167.43, 162.82, 151.53, 150.00, 148.42, 139.19, 134.48, 125.49, 124.35, 123.25, 122.97, 116.38, 115.89, 114.61, 109.84, 96.36, 55.94 ppm. *Anal* Calcd. for C_18_H_15_N_3_O_5_, C, 61.19, H, 4.28, N, 11.89 found C, 60.98, H, 4.08, N, 10.03.

### α-glucosidase inhibition assay

α-glucosidase enzyme (EC3.2.1.20, *Saccharomyces cerevisiae*, 20 U/mg) and substrate (p-nitrophenyl glucopyranoside) were purchased from Sigma-Aldrich. 1 mg of α-glucosidase was dissolved in potassium phosphate buffer (50 mM, pH = 6.8) to obtain the initial activity of 1 U/ml. Then, 20 µl of this enzyme solution was incubated with 135 µl of potassium phosphate buffer and 20 µl of test compound at various concentrations in DMSO. After 10 min incubation at 37 °C, 25 µl of the substrate at a final concentration of 4 mM was added to the mixture and allowed to incubate at 37 °C for 20 min. Then, the change in absorbance was measured at 405 nm spectroscopically. DMSO (10% final concentration) as control and acarbose as the standard inhibitor were used. The percentage of inhibition for each entry was calculated by using the following formula:

$$\% \,{\text{ Inhibition}}\, = \,\left[ {\left( {{\text{Abs}}\,{\text{control}} - {\text{Abs}}\,{\text{sample}}} \right)/{\text{Abs}}\,{\text{control}}} \right]\, \times \,{1}00.$$

IC_50_ values were obtained from the nonlinear regression curve using the Logit method^28,29^.

### Enzyme kinetic studies

The mode of inhibition of the most active compounds **6l** and **6m** identified with the lowest IC_50_, was investigated against an α-glucosidase activity with different concentrations of *p*-nitrophenyl *α*-d-glucopyranoside (1–12 mM) as substrate in the absence and presence of **6l **and **6m** at different concentrations. A Lineweaver–Burk plot was generated to identify the type of inhibition and the Michaelis–Menten constant (*K*_m_) value was determined from the plot between the reciprocal of the substrate concentration (1/[S]) and reciprocal of enzyme rate (1/V) over various inhibitors concentrations^30,31^.

### Molecular docking

The molecular docking studies were performed using the Maestro Molecular Modeling platform (version 10.5) by Schrödinger, LLC. The homology model structure of a-glucosidase was obtained according to the previously reported procedure^32,33^. The protein is then prepared using a protein preparation wizard so that co-crystallized ligands and all water molecules were removed, the missing side chains and loops were filled using the prime tool, and PROPKA assigned H-bonds at pH: 7.4. To prepare the ligands, the 2D structures of the ligands were drawn in ChemDraw and converted into SDF files and subjected to ligprep module. Ligands were prepared by OPLS_2005 force field using EPIK. The grid box was generated for each binding site using entries with a box size of 25 A, the derivative was docked on binding sites using induced-fit docking, reporting 10 poses per ligand to form the final complex.

### Molecular dynamic simulation

The molecular simulation was performed using the Desmond v5.3 (Schrödinger 2018‐4 suite). To build the system for MD simulation, the protein-ligands complexes were solvated with SPC explicit water molecules and placed in the center of an orthorhombic box of appropriate size in the periodic boundary condition. Sufficient counter‐ions and a 0.15 M solution of NaCl were also utilized to neutralize the system and to simulate the real cellular ionic concentrations, respectively. The MD protocol involved minimization, pre-production, and finally production MD simulation steps^7^. In the minimization procedure, the entire system was allowed to relax for 2500 steps by the steepest descent approach. Then the temperature of the system was raised from 0 to 300 K with a small force constant on the enzyme to restrict any drastic changes. MD simulations were performed via NPT (constant number of atoms, constant pressure i.e. 1.01325 bar, and constant temperature i.e. 300 K) ensemble. The Nose–Hoover chain method was used as the default thermostat with 1.0 ps interval and Martyna–Tobias–Klein as the default barostat with 2.0 ps interval by applying isotropic coupling style. Long‐range electrostatic forces were calculated based on the particle‐mesh‐based Ewald approach with the cut‐off radius for Columbia forces set to 9.0 Å. Finally, the system was subjected to produce MD simulations for 30 ns for each protein–ligand complex. The dynamic behavior and structural changes of the systems were analyzed by the calculation of the RMSD and RMSF^24^.

#### Prime MM-GBSA

The ligand binding energies (ΔG_Bind_) were calculated for 6l and 6m using Prime-Molecular mechanics/generalized Born surface area (MM GBSA) modules (Schrödinger LLC 2018) based on the following equation

$$\Delta {\text{G}}_{{{\text{Bind}}}} = {\text{E}}_{{{\text{complex}}}} {-}{\text{ E}}_{{{\text{Protein}} - }} {\text{E}}_{{{\text{Ligand}}}} ,$$

where ΔG_Bind_ is the calculated relative free energy which includes both ligand and receptor strain energy. E_Complex_ is the MM-GBSA energy of the minimized complex, and E_protein_ is the MM-GBSA energy of relaxed protein after separating it from the ligand. E_Ligand_ is the MM-GBSA energy of the ligand after removing it from the complex and allowing it to relax^34^.

## Supplementary Information

Supplementary Information.

## Data Availability

The datasets used and/or analysed during the current study available from the corresponding author on reasonable request.
