# Supplementary material for: Synthesis, in vitro α-glucosidase inhibitory activities, and molecular dynamic simulations of novel 4-hydroxyquinolinone-hydrazones as potential antidiabetic agents
Source: Sci Rep. 2023 Apr 18;13:6304. doi: 10.1038/s41598-023-32889-7 (PMC10113378; doi:10.1038/s41598-023-32889-7)
Supplement: Supplementary file 1 — Supplementary Information. [file 41598_2023_32889_MOESM1_ESM.docx]

**Table S1.** Glide score of all derivatives against homology-modeled-glucosidase of S. cerevisiae

| **Compounds** | **Glide score (**Kcal/mol**)** | **Compounds** | **Glide score (**Kcal/mol**)** |
| --- | --- | --- | --- |
| **6a** | -7.03 | **6i** | -5.76 |
| **6b** | -8.08 | **6j** | -7.45 |
| **6c** | -7.53 | **6k** | -7.29 |
| **6d** | -7.93 | **6l** | -8.29 |
| **6e** | -5.26 | **6m** | -8.14 |
| **6f** | -6.23 | **6n** | -7.38 |
| **6g** | -8.15 | **6o** | -5.22 |
| **6h** | -8.22 |  |  |

**(*E*)-*N'*-benzylidene-4-hydroxy-2-oxo-1,2-dihydroquinoline-3-carbohydrazide**


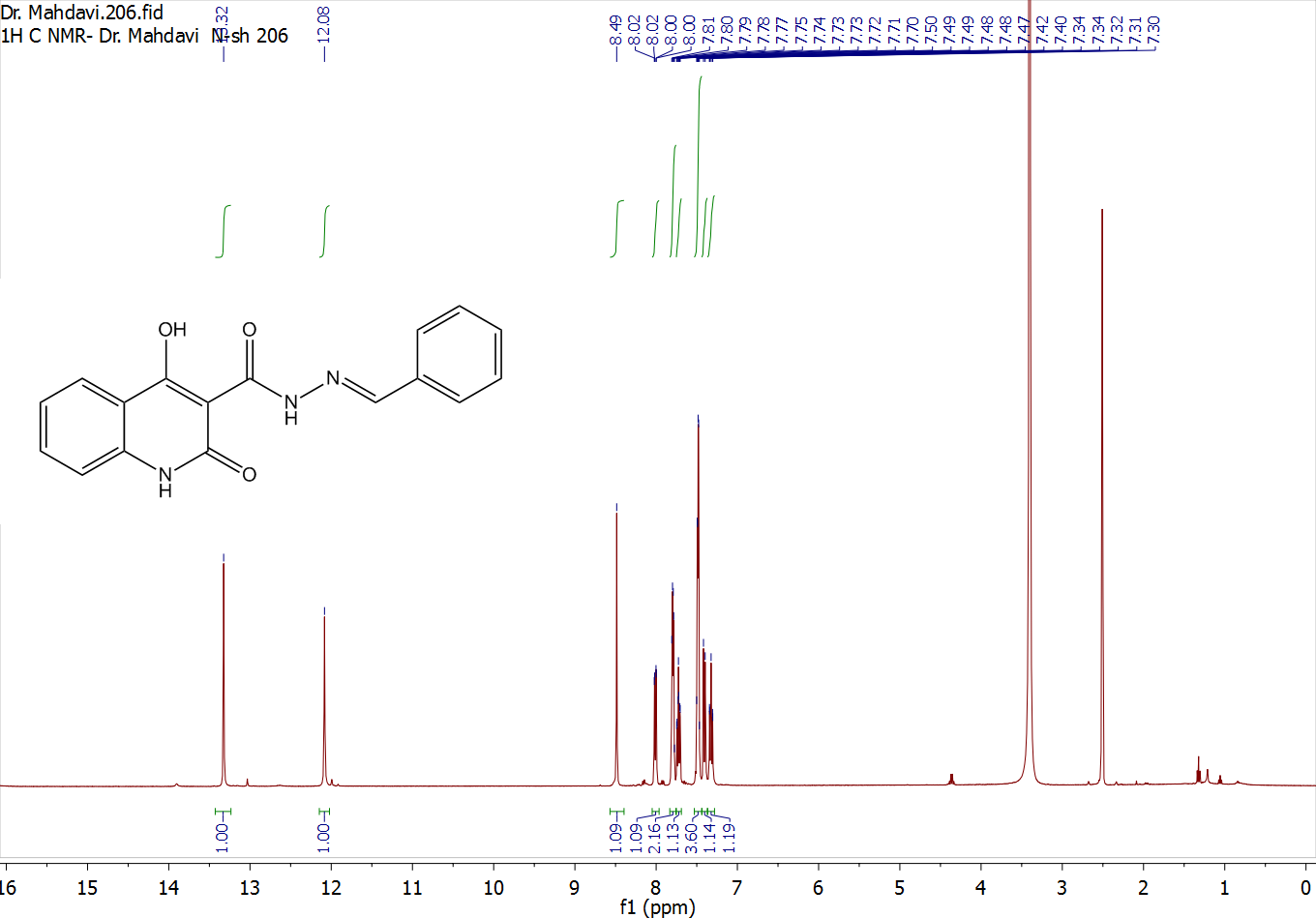


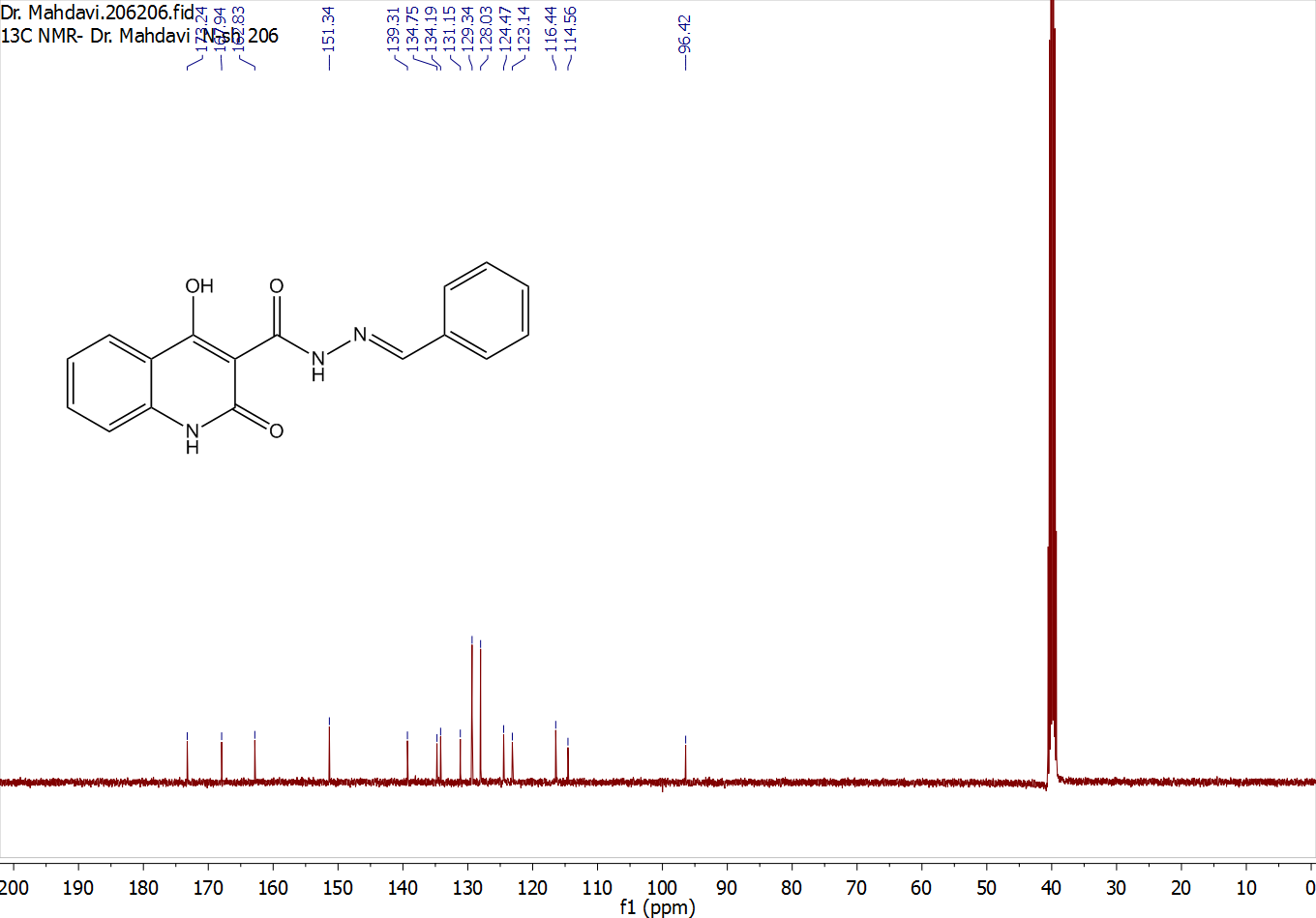


**(*E*)-*N'*-(2-fluorobenzylidene)-4-hydroxy-2-oxo-1,2-dihydroquinoline-3-carbohydrazide**


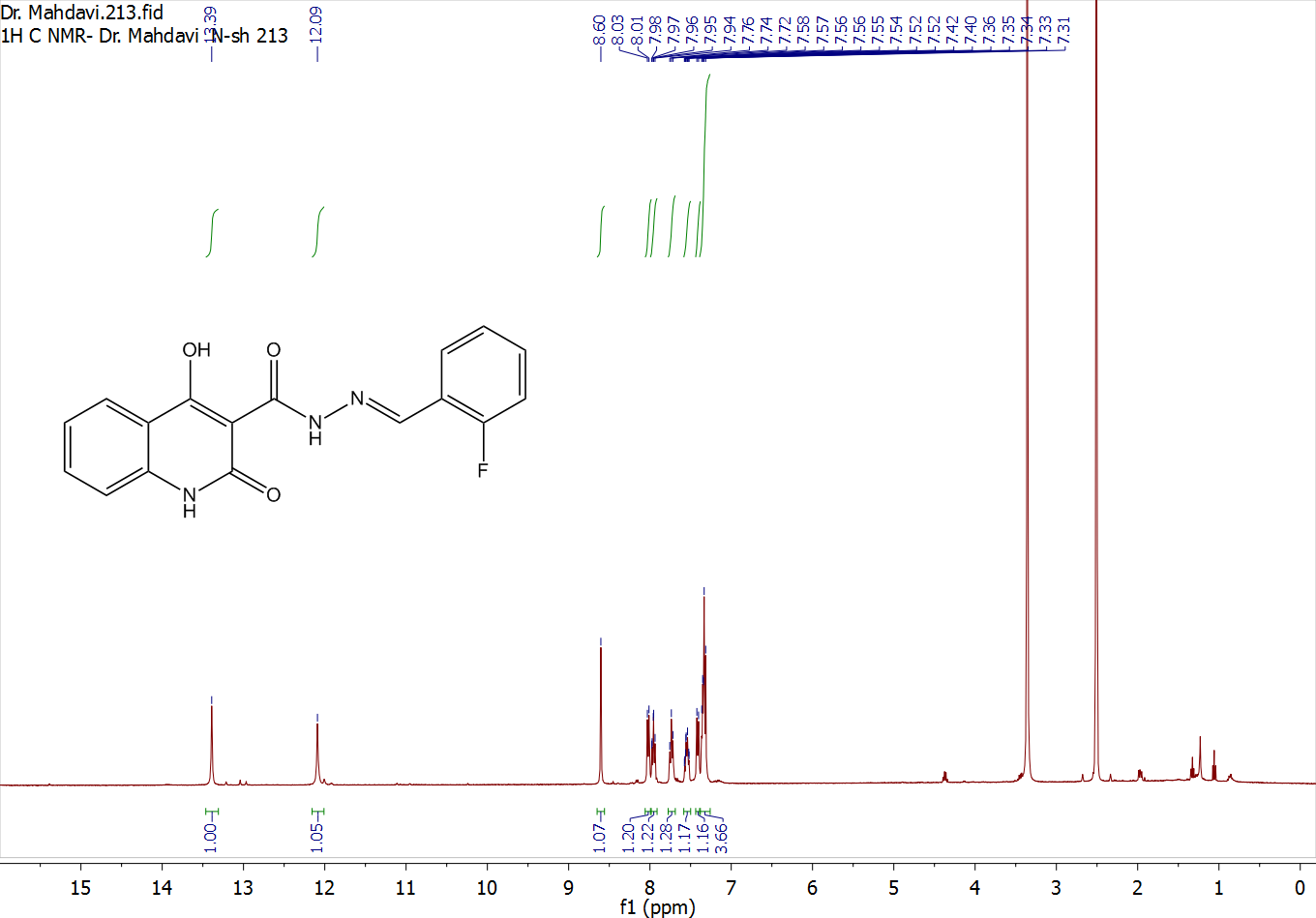


**
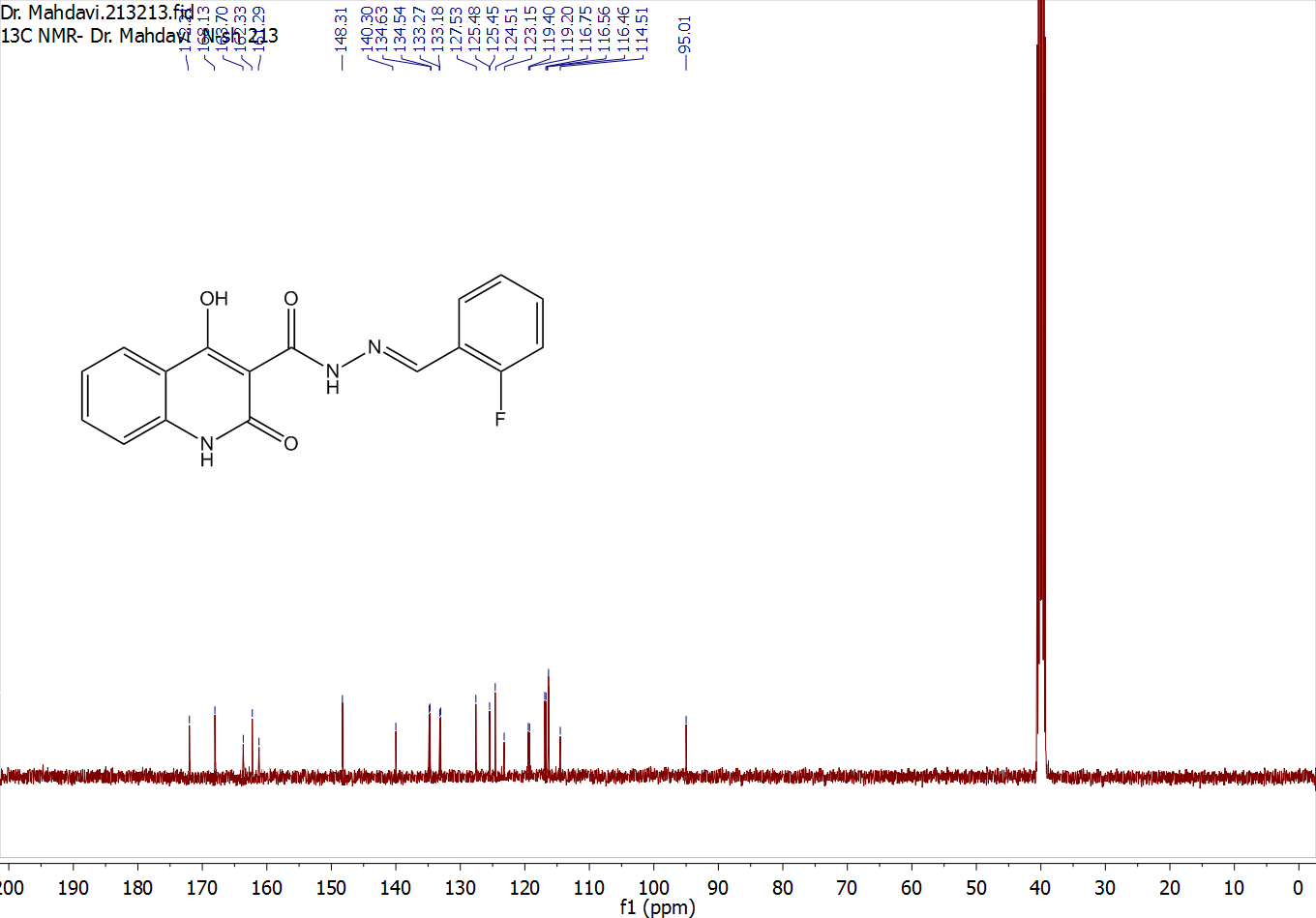
**

**(*E*)-*N'*-(4-fluorobenzylidene)-4-hydroxy-2-oxo-1,2-dihydroquinoline-3-carbohydrazide**


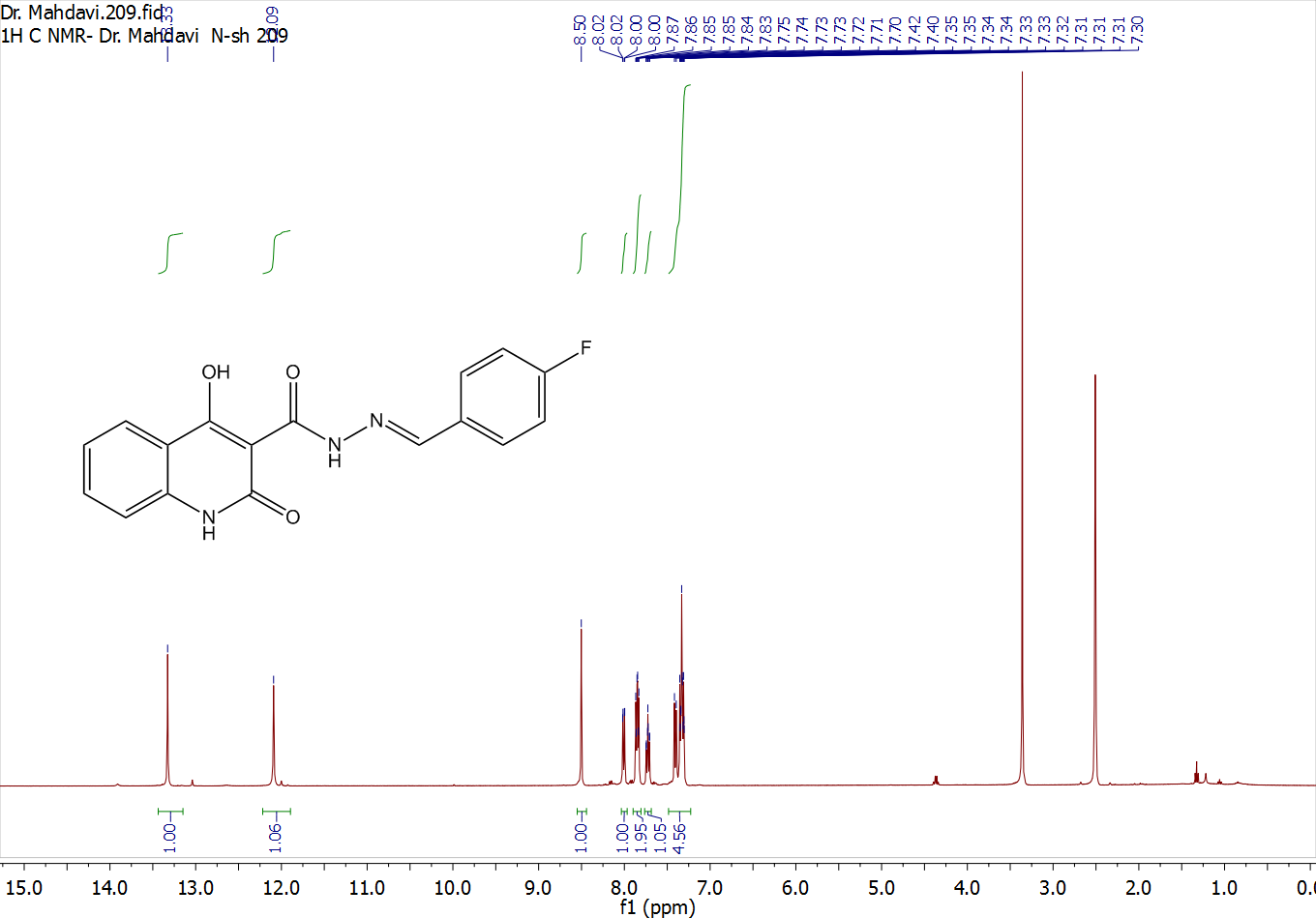


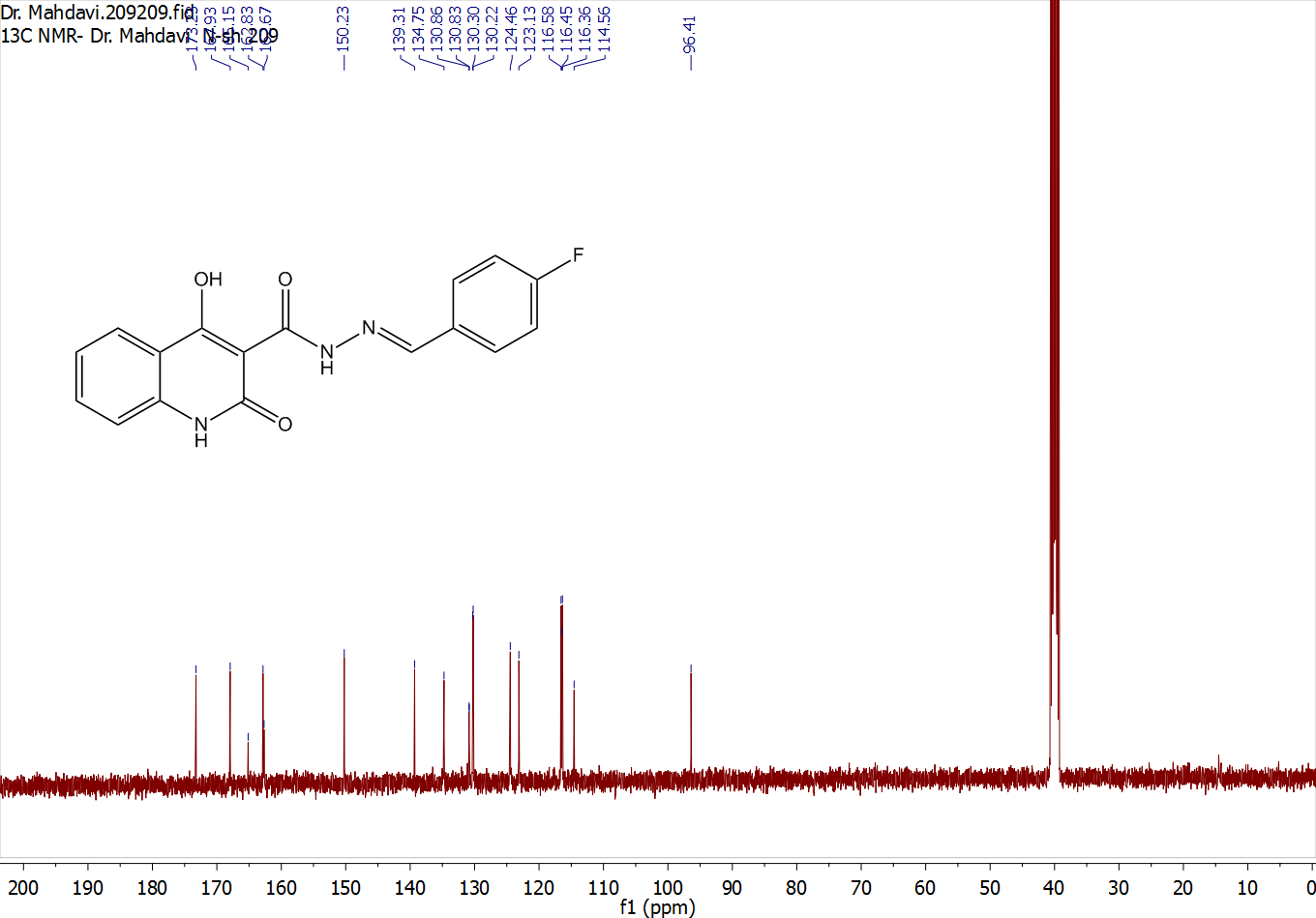


**(*E*)-*N'*-(4-chlorobenzylidene)-4-hydroxy-2-oxo-1,2-dihydroquinoline-3-carbohydrazide**
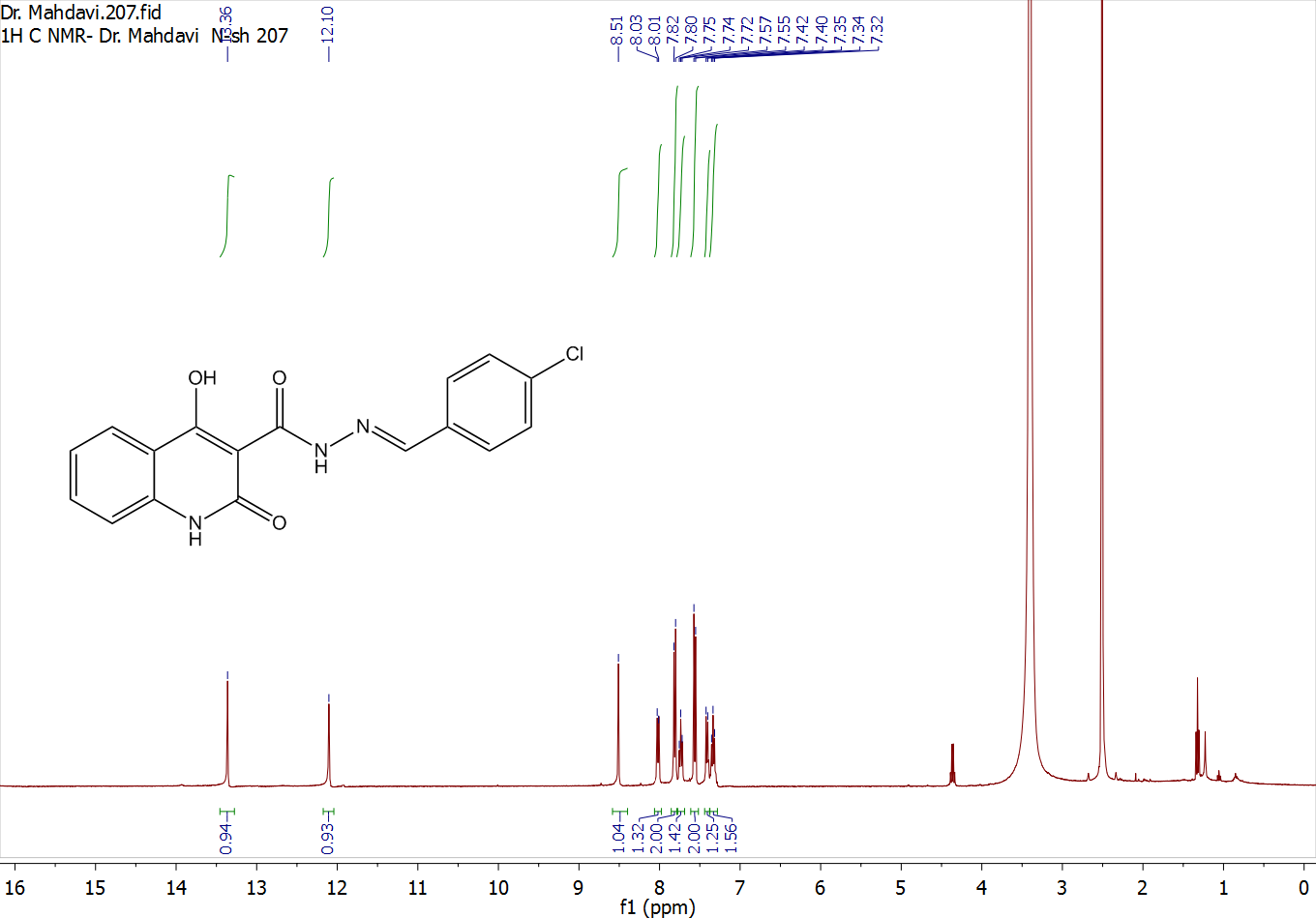


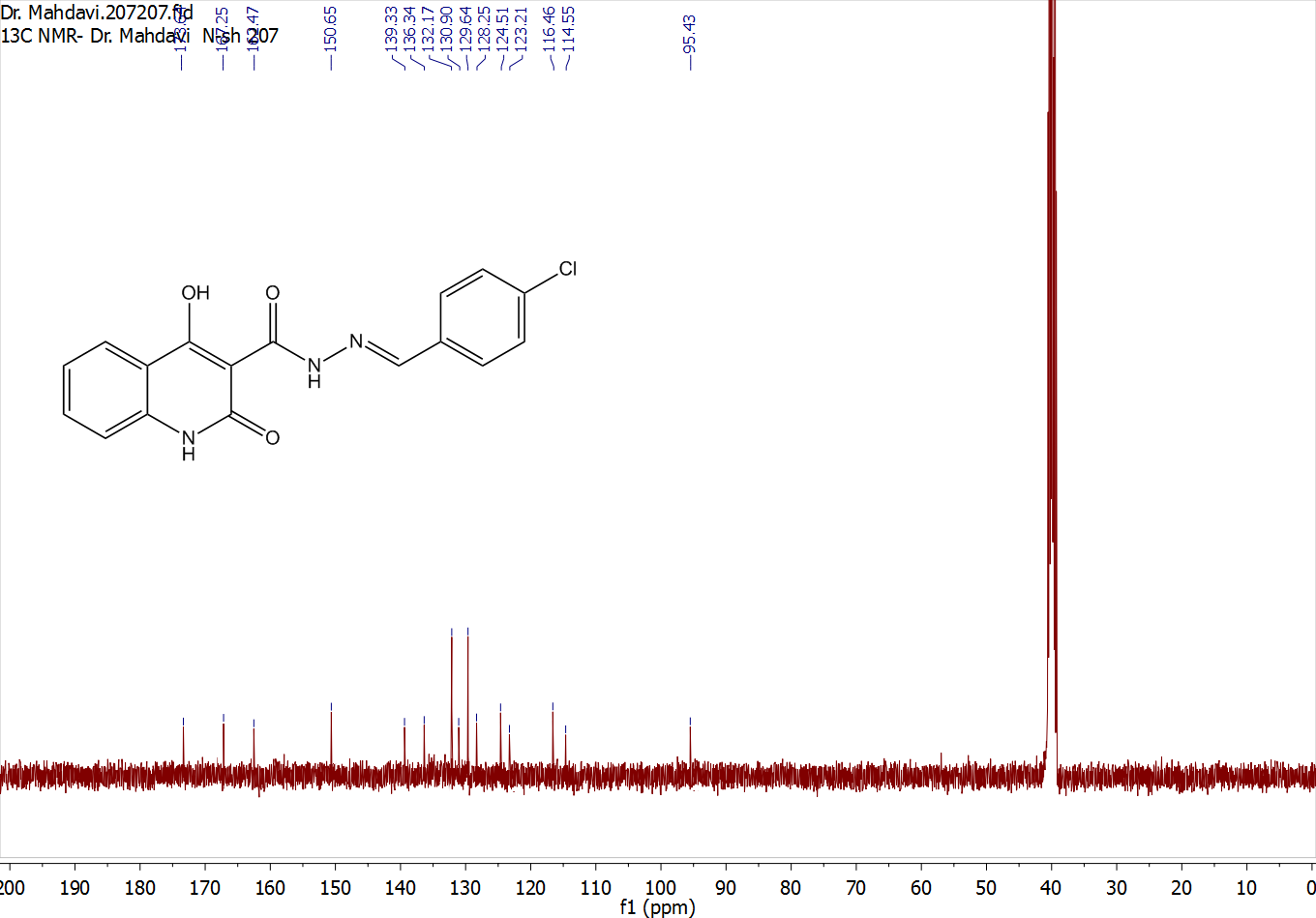


**(*E*)-*N'*-(3-bromobenzylidene)-4-hydroxy-2-oxo-1,2-dihydroquinoline-3-carbohydrazide**


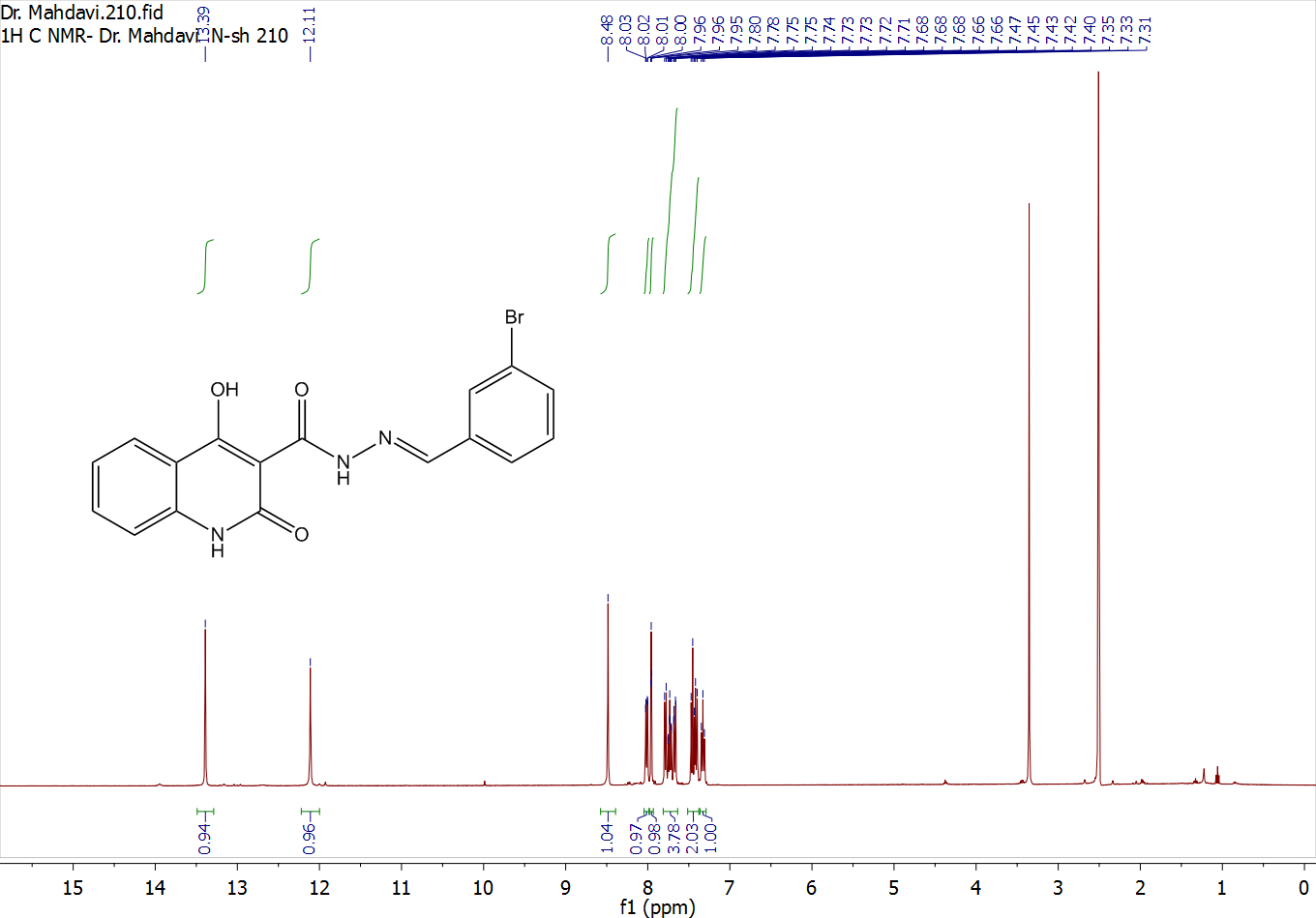


**
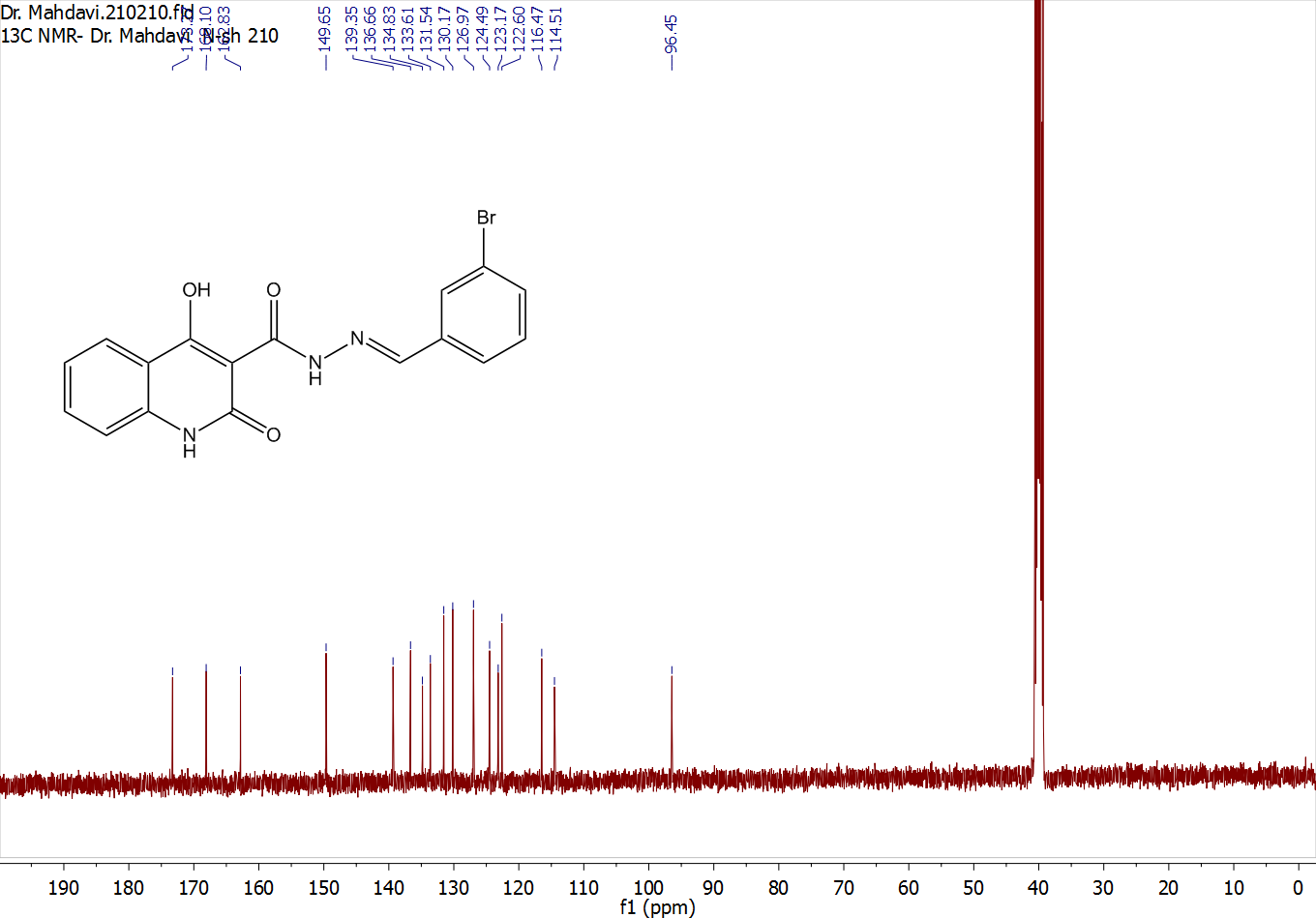
**

**(*E*)-*N'*-(4-bromobenzylidene)-4-hydroxy-2-oxo-1,2-dihydroquinoline-3-carbohydrazide**


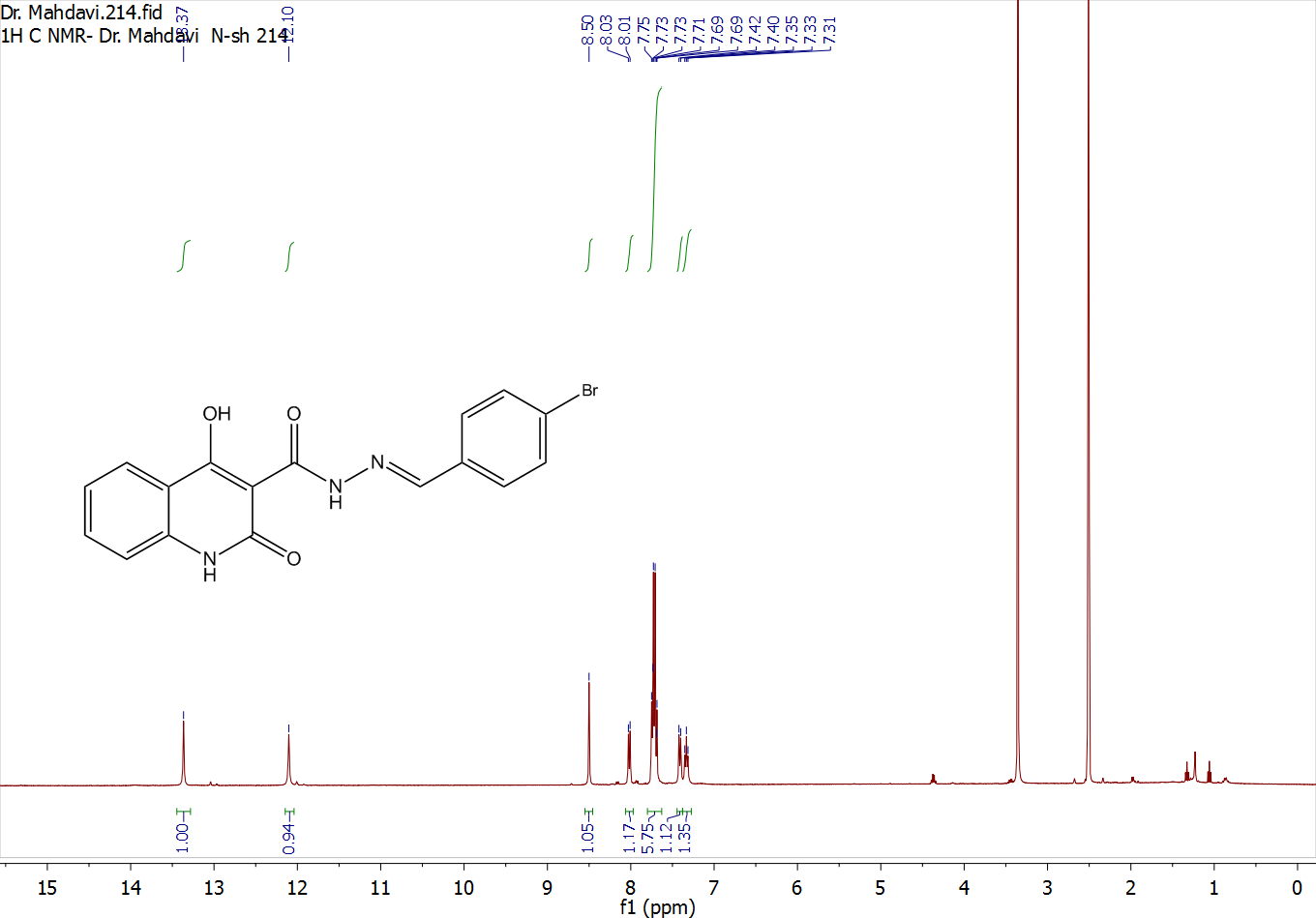


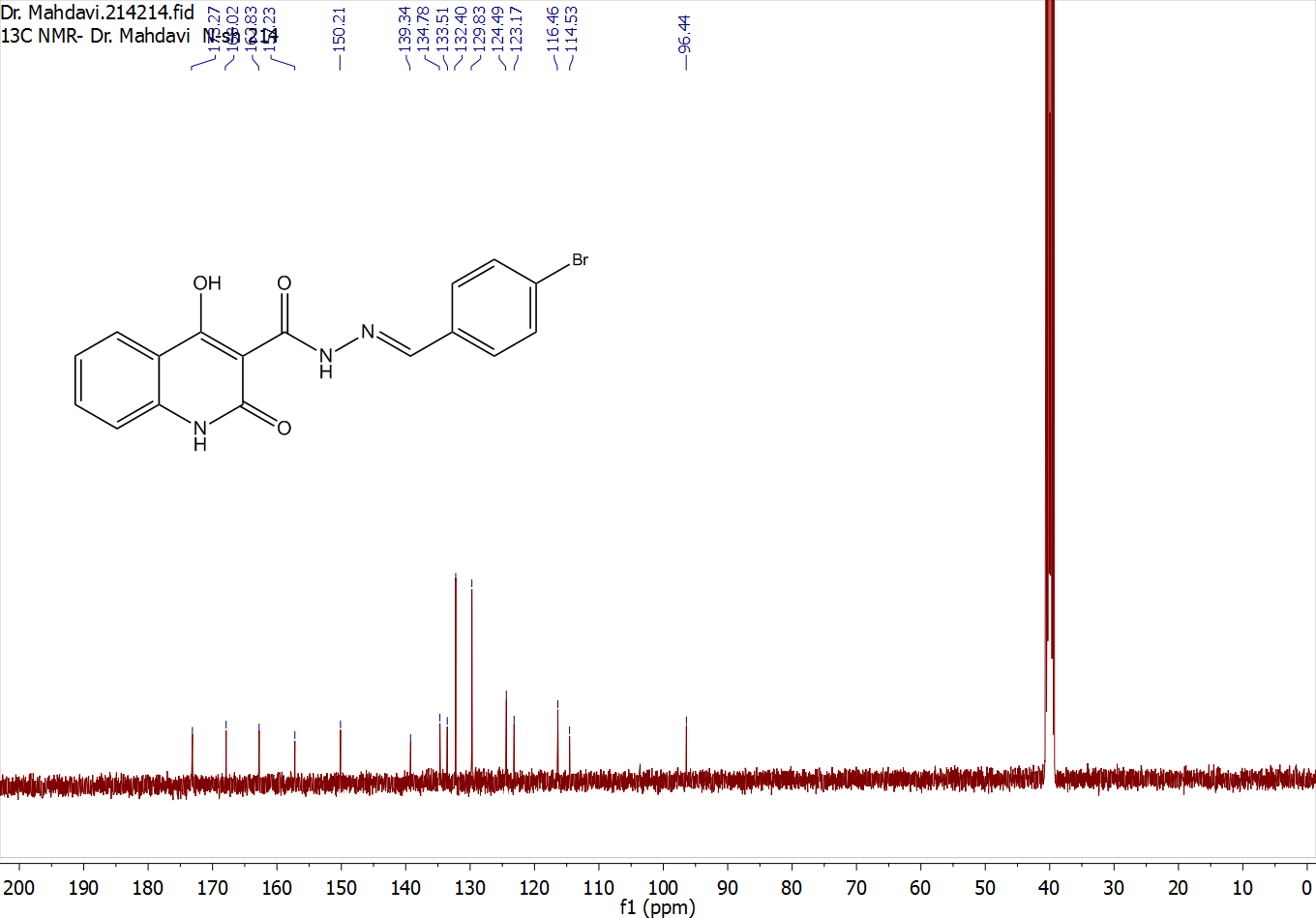


**(*E*)-4-hydroxy-*N'*-(4-nitrobenzylidene)-2-oxo-1,2-dihydroquinoline-3-carbohydrazide**


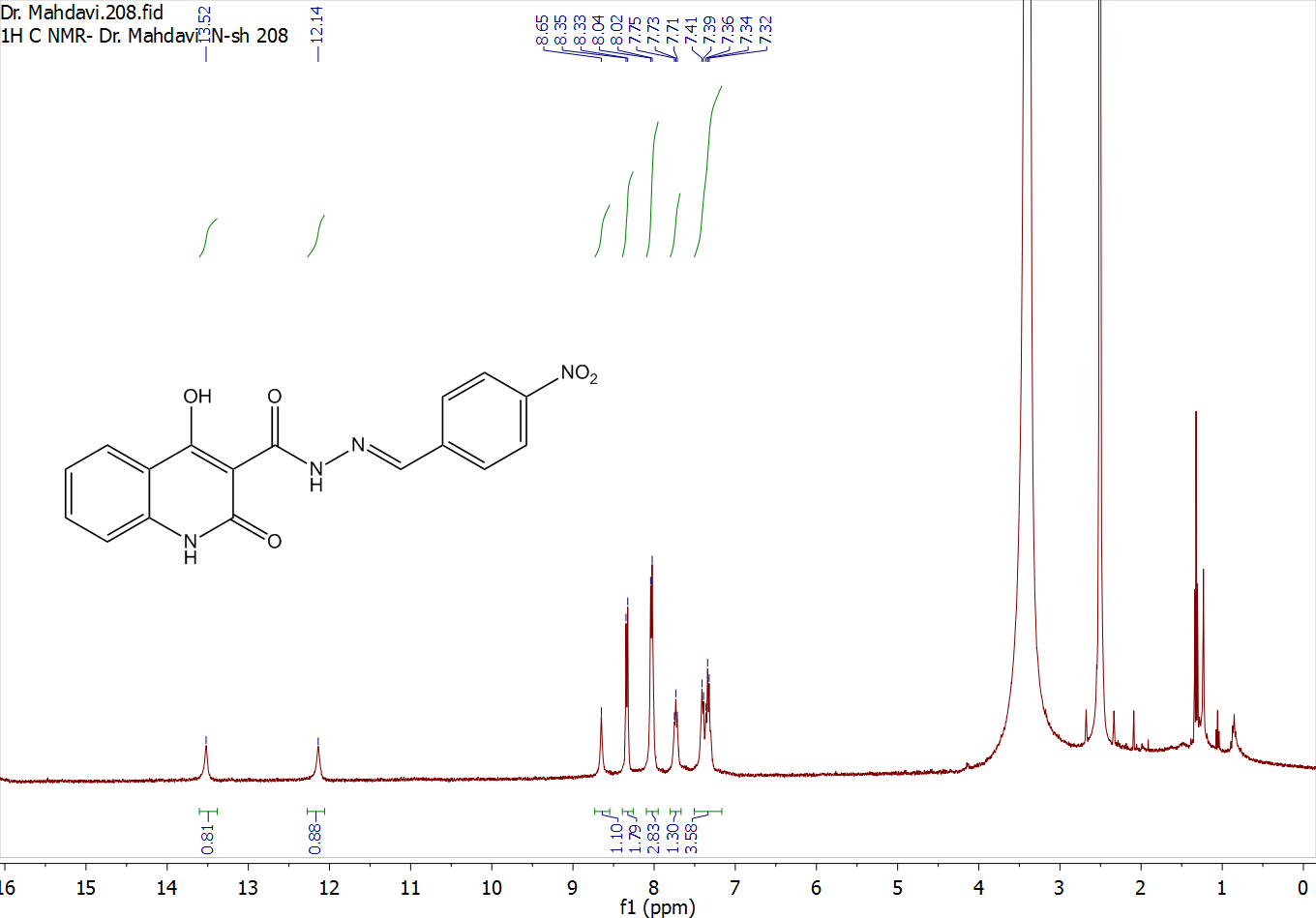


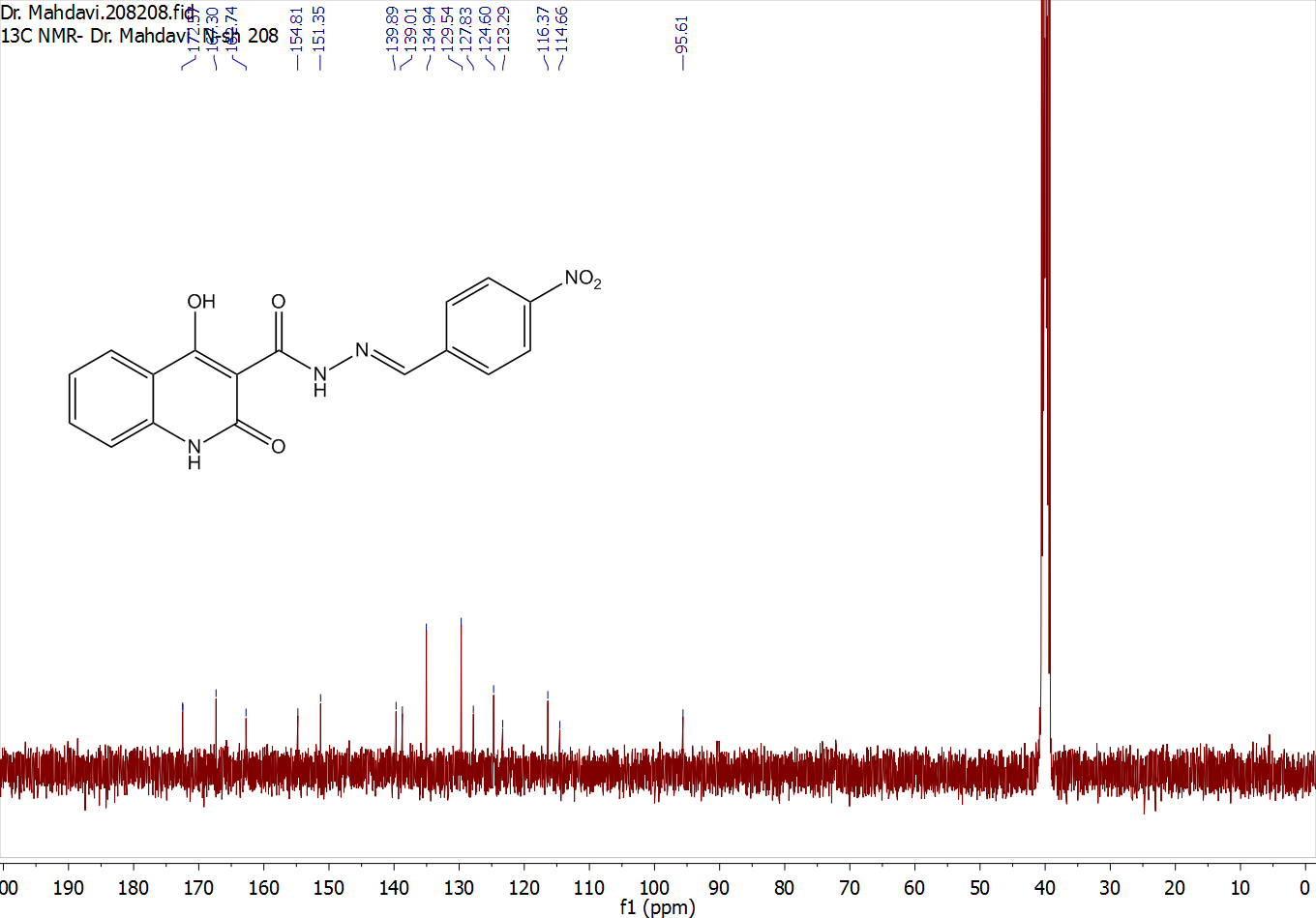


**(*E*)-4-hydroxy-*N'*-(4-methylbenzylidene)-2-oxo-1,2-dihydroquinoline-3-carbohydrazide**


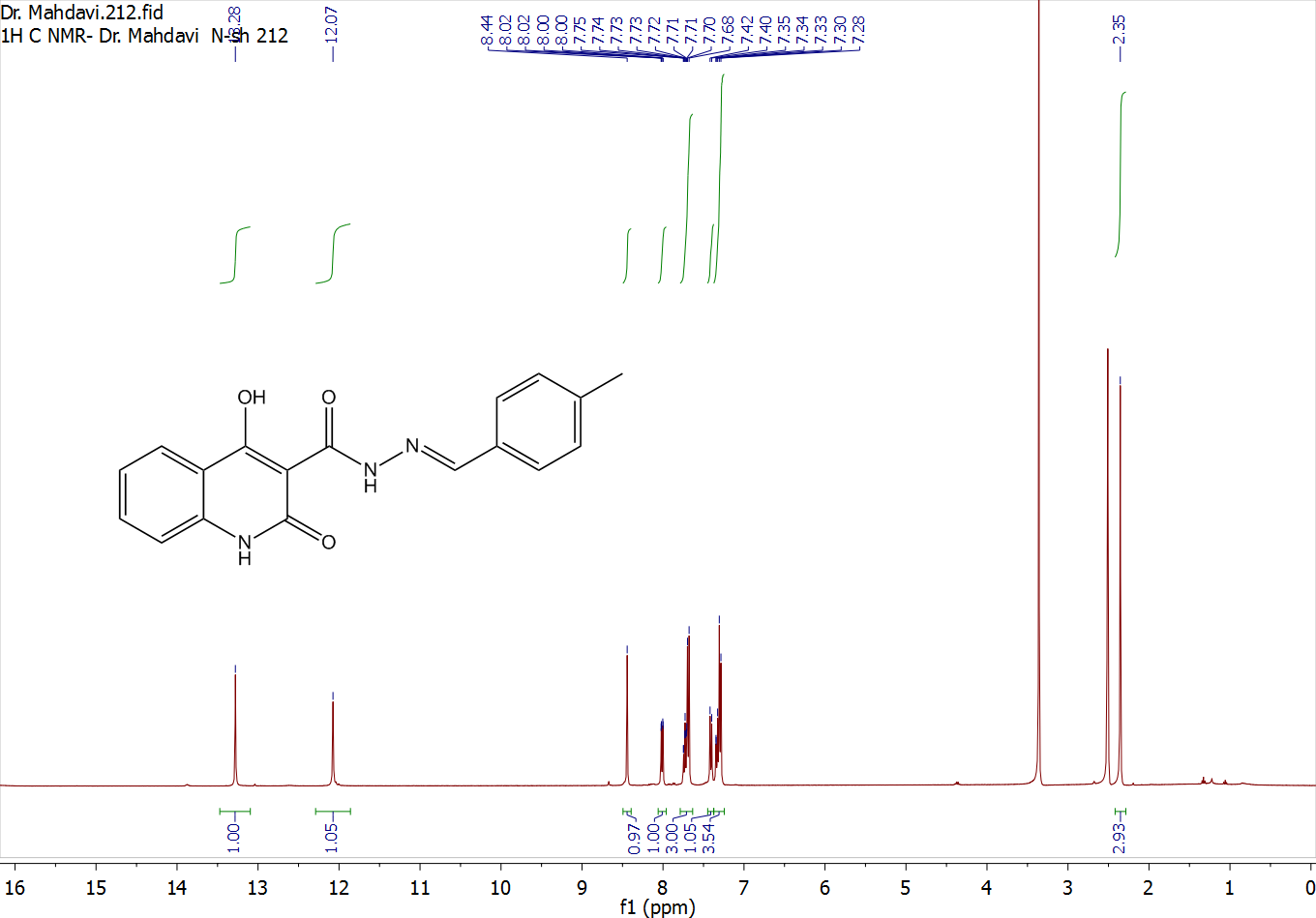


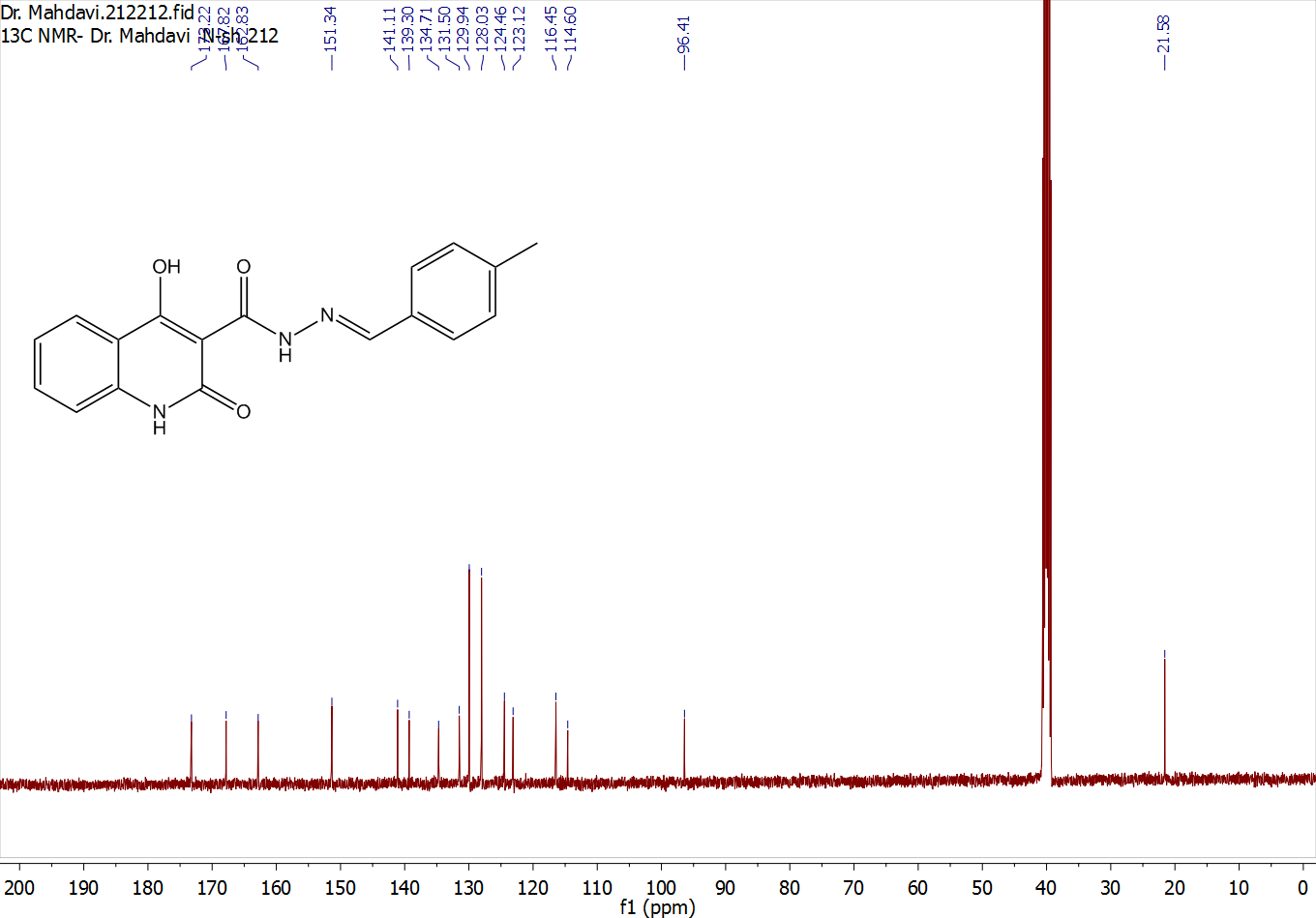


**(*E*)-4-hydroxy-*N'*-(4-methoxybenzylidene)-2-oxo-1,2-dihydroquinoline-3-carbohydrazide**


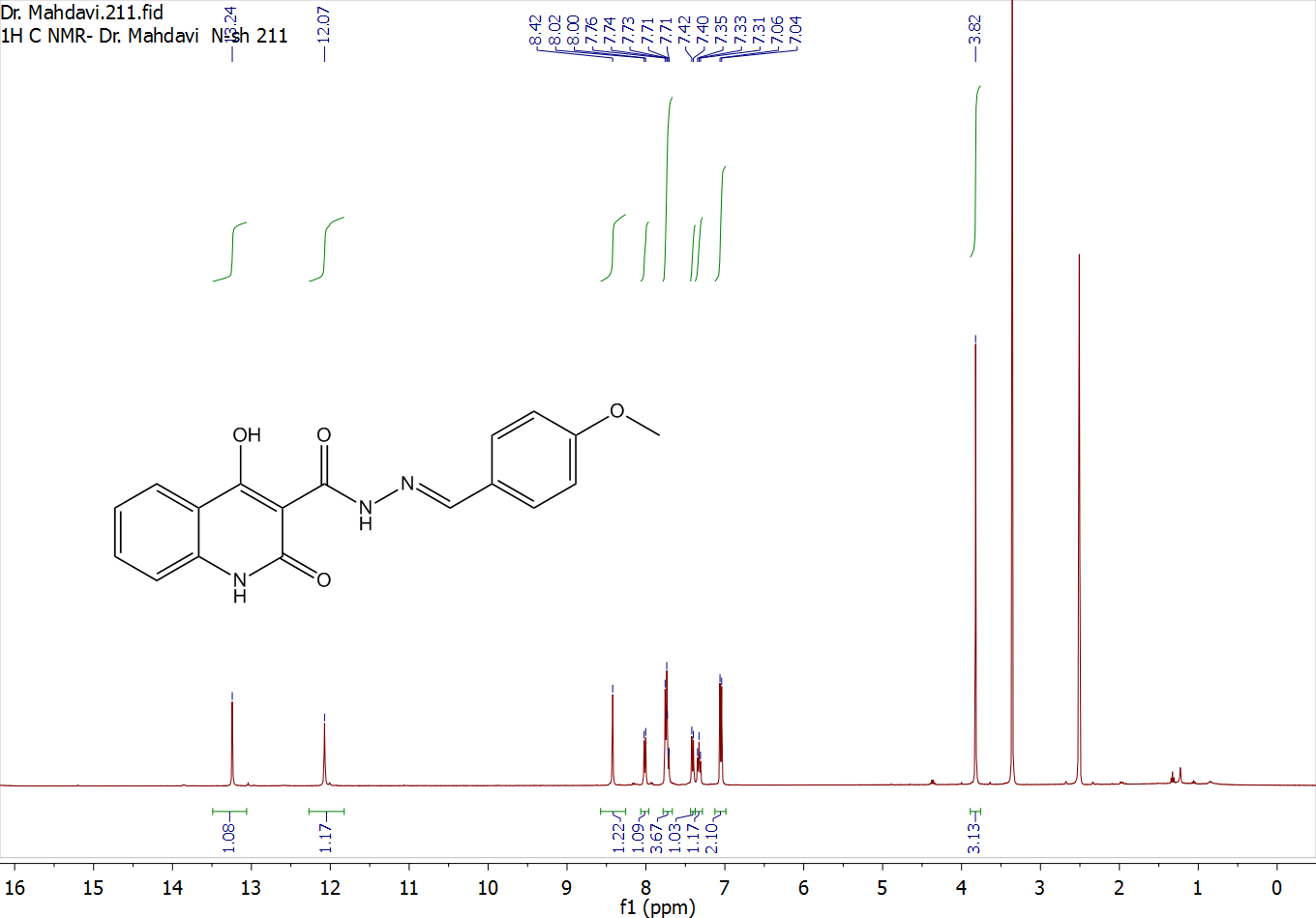


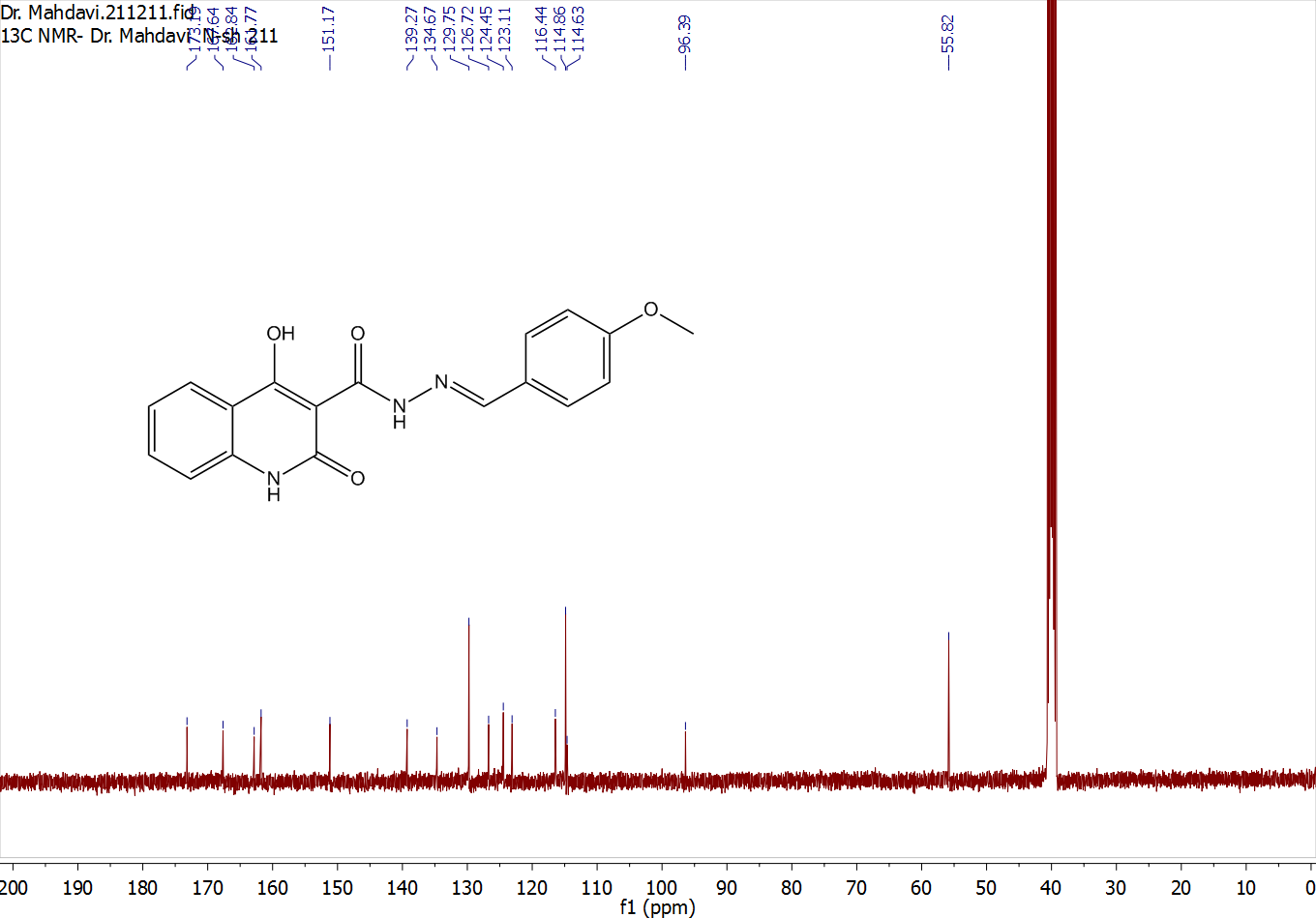


**(*E*)-4-hydroxy-*N'*-(2-hydroxybenzylidene)-2-oxo-1,2-dihydroquinoline-3-carbohydrazide**


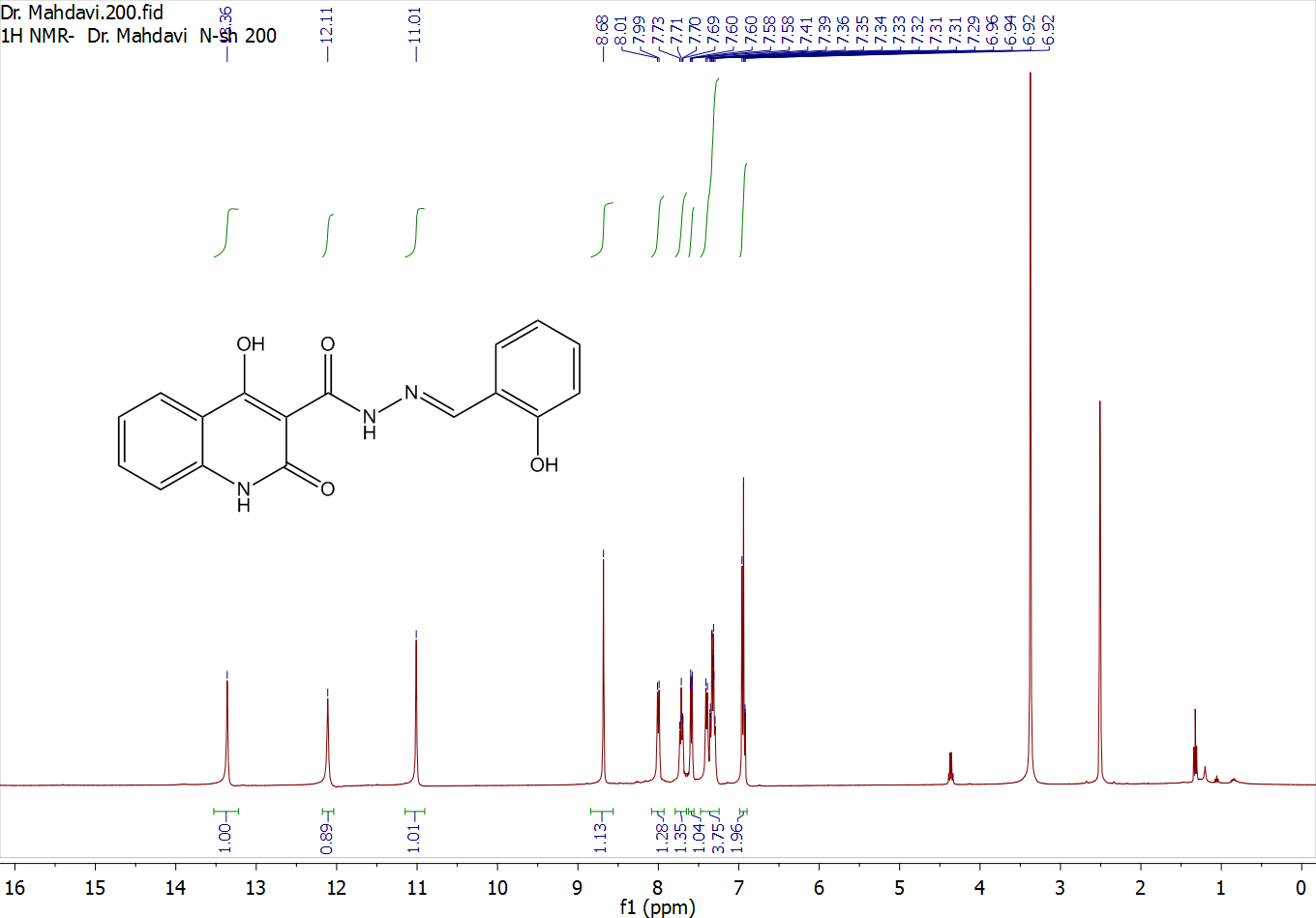


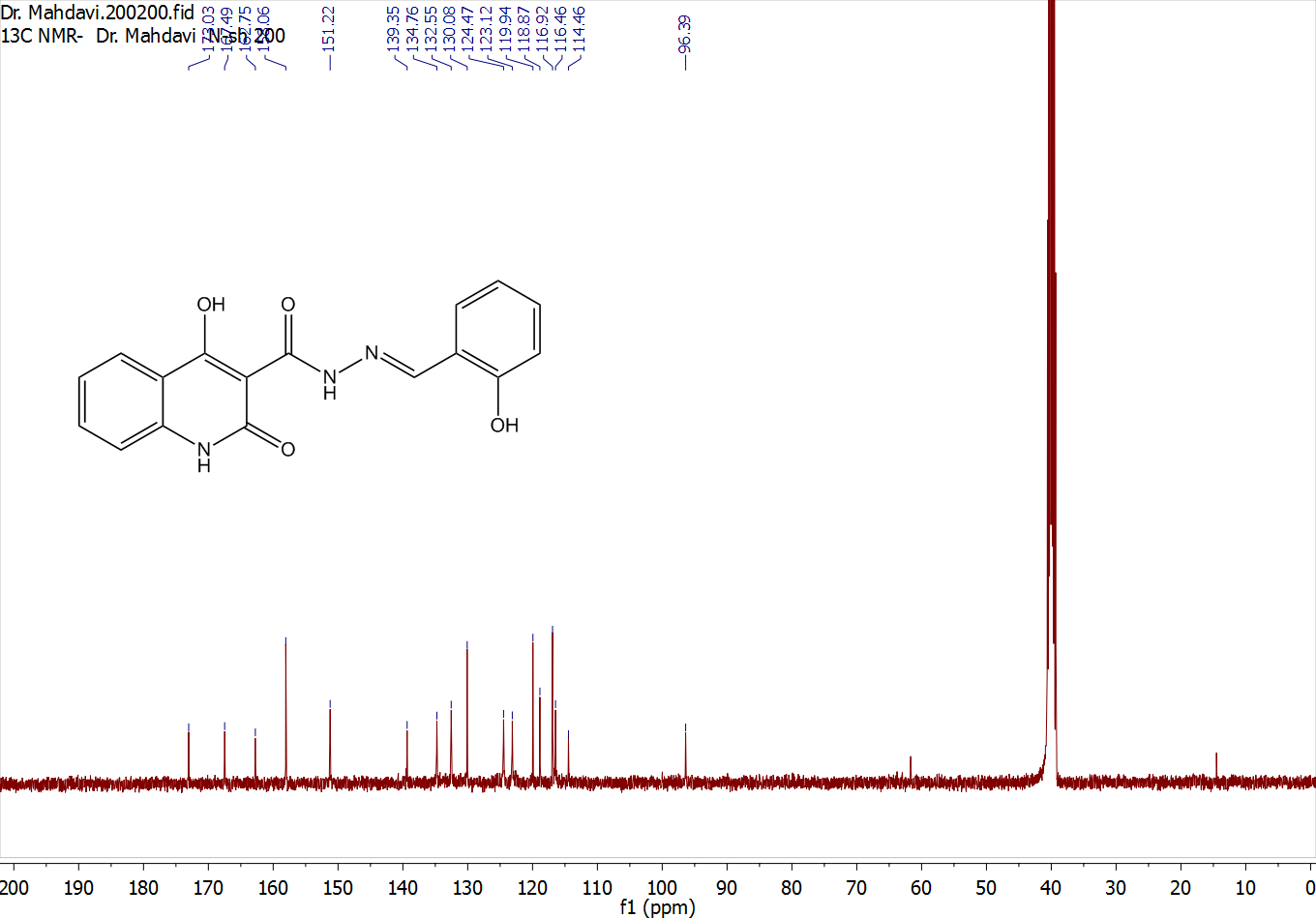


**(*E*)-4-hydroxy-*N'*-(3-hydroxybenzylidene)-2-oxo-1,2-dihydroquinoline-3-carbohydrazide**


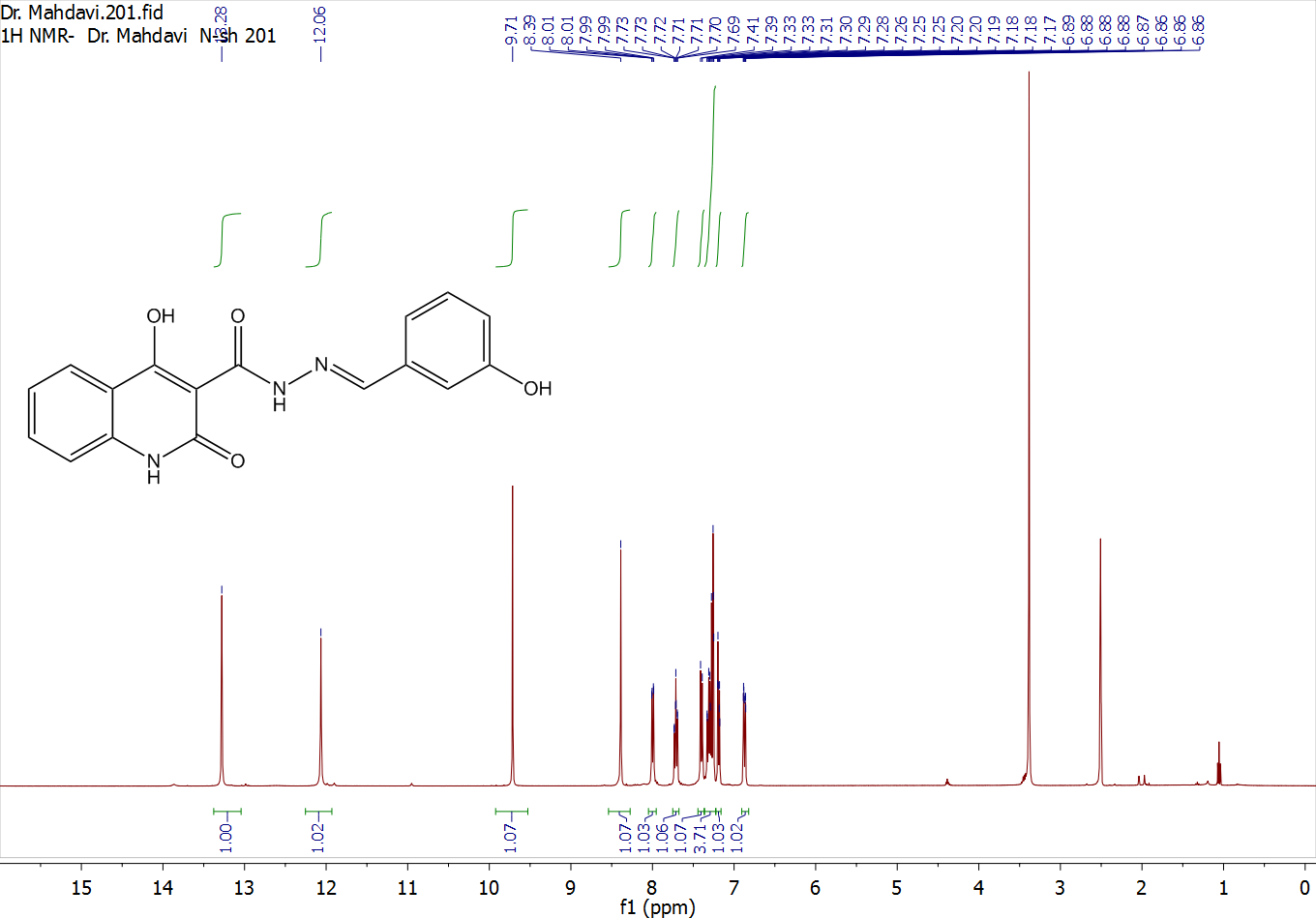


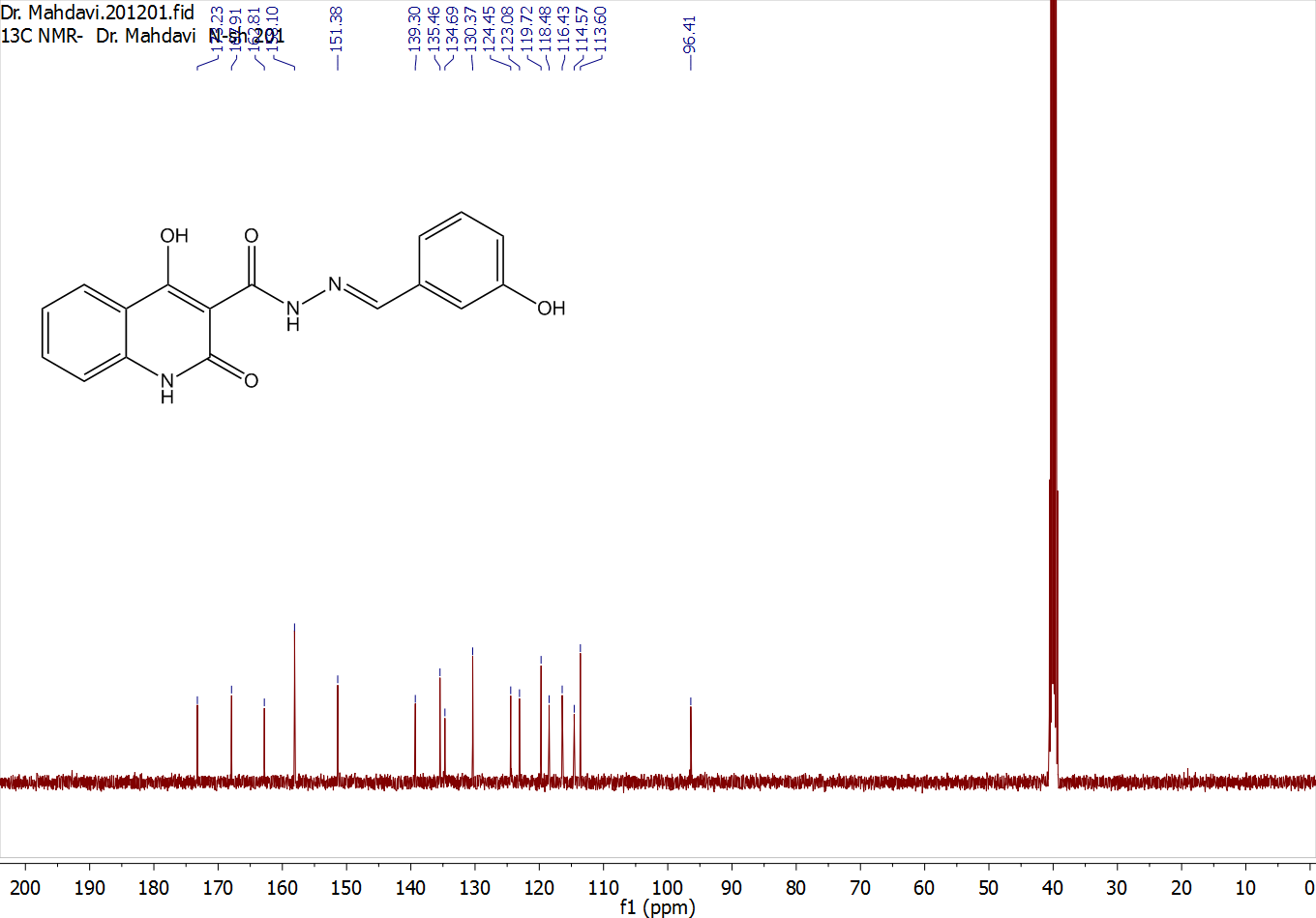


**(*E*)-*N'*-(5-bromo-2-hydroxybenzylidene)-4-hydroxy-2-oxo-1,2-dihydroquinoline-3-carbohydrazide**


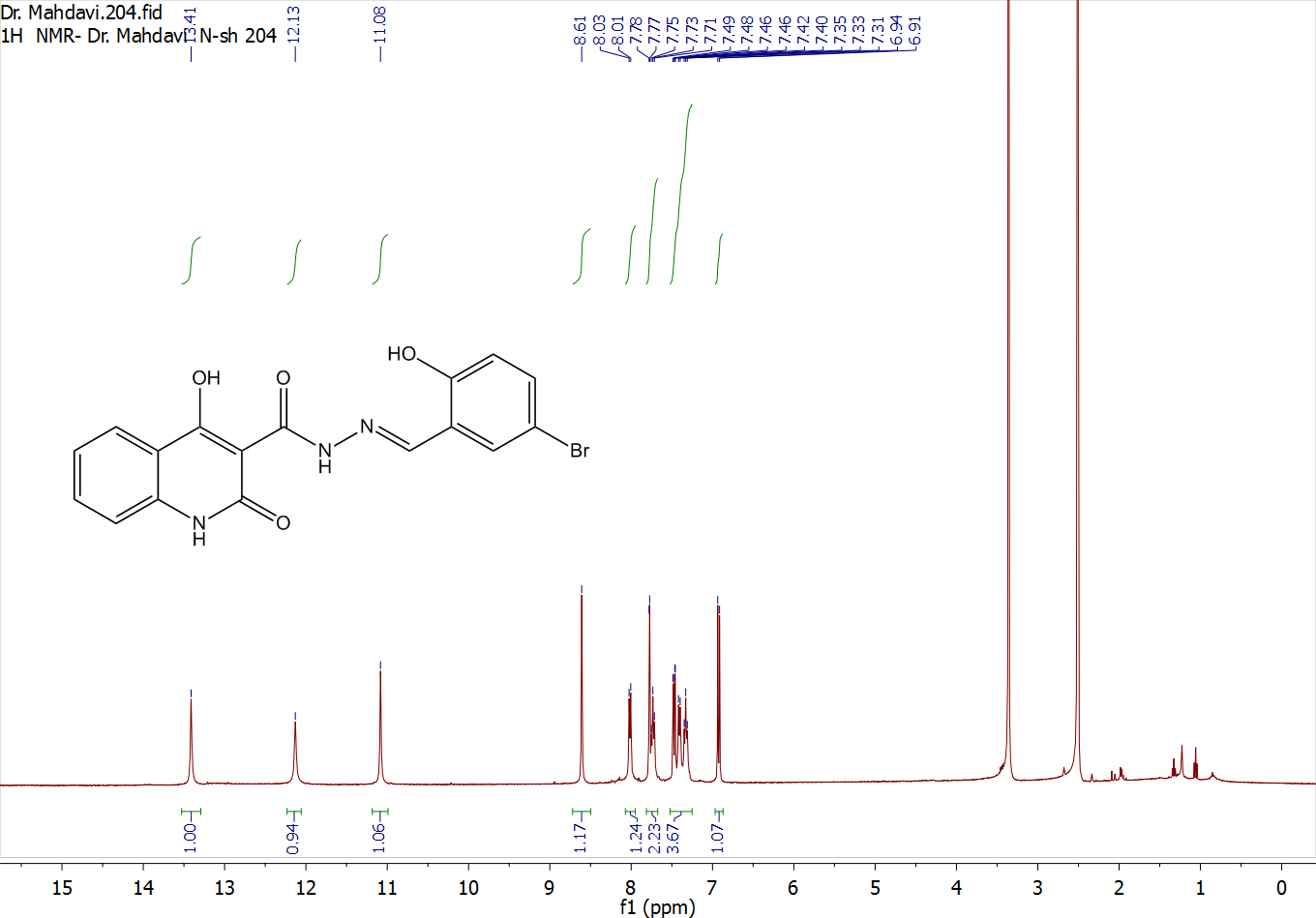


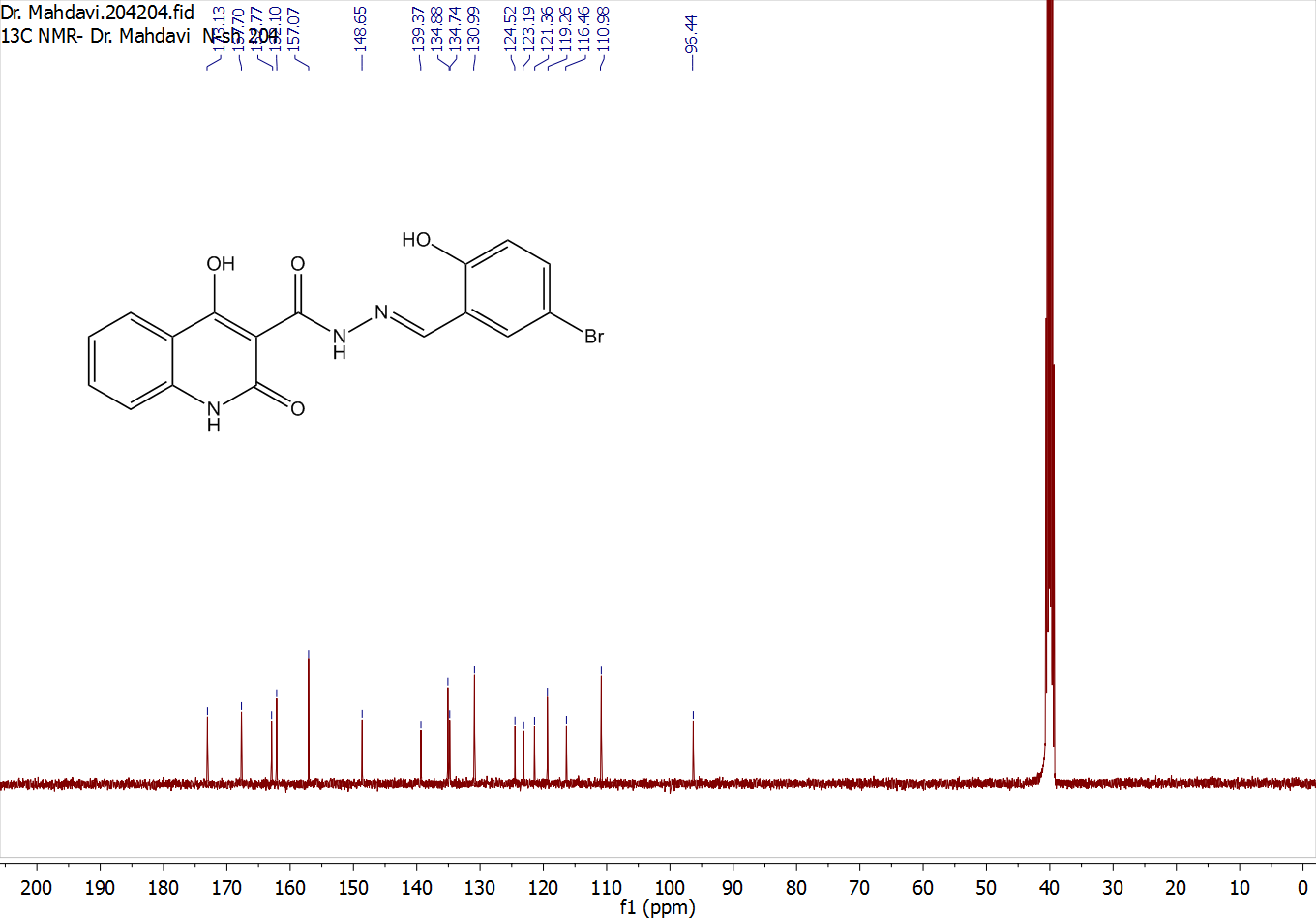


**(*E*)-4-hydroxy-*N'*-(2-hydroxy-5-nitrobenzylidene)-2-oxo-1,2-dihydroquinoline-3-carbohydrazide**


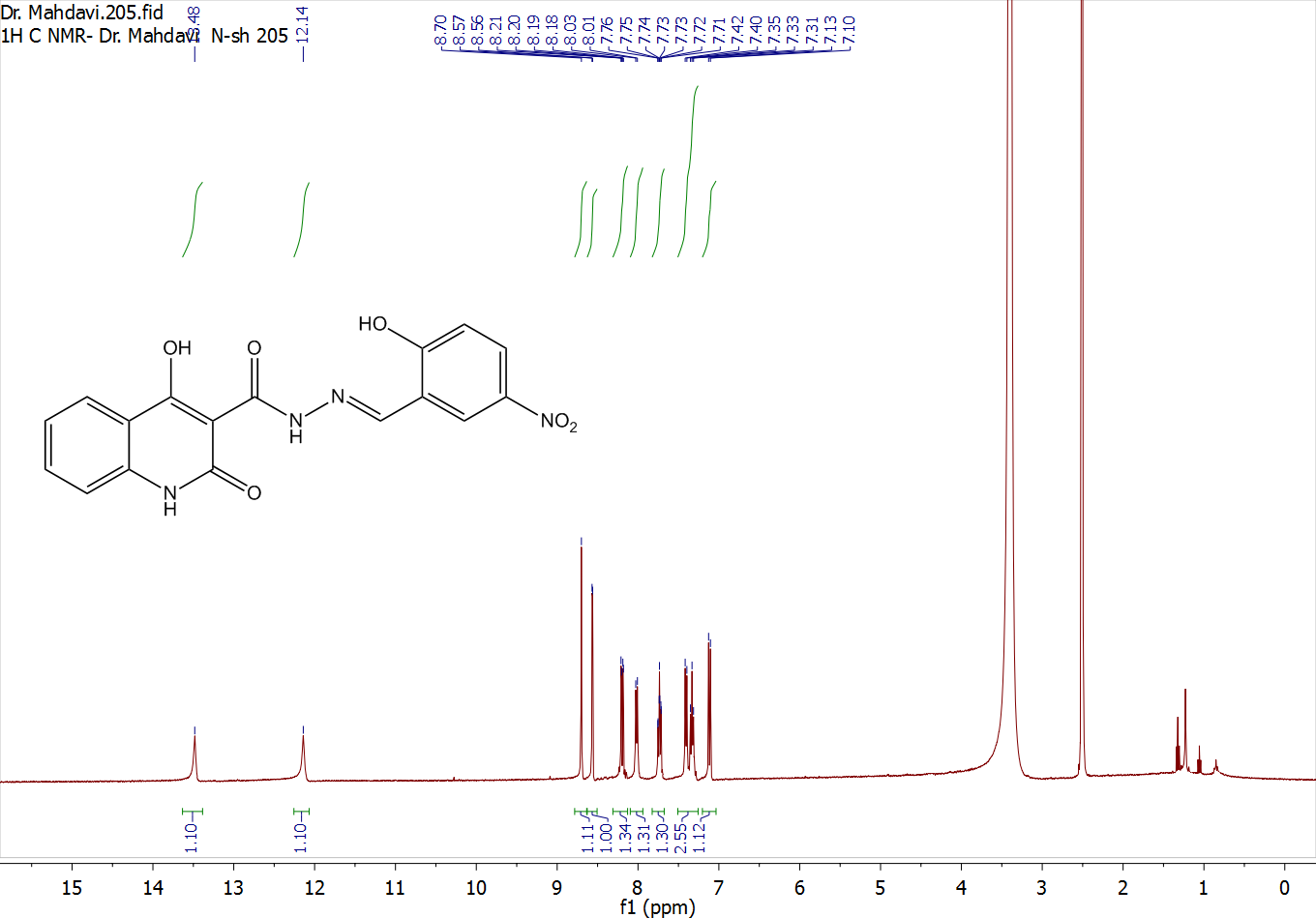


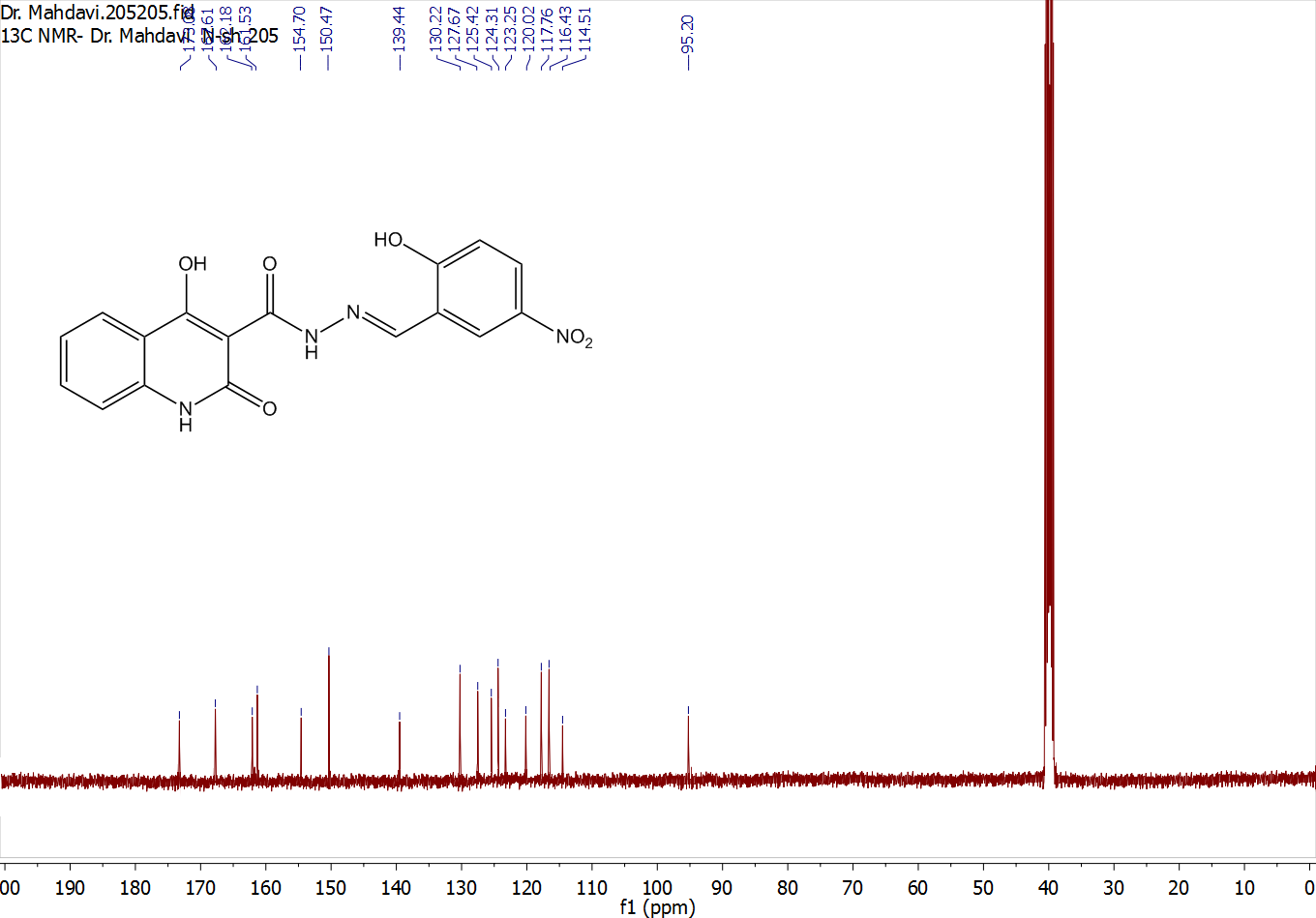


**(*E*)-*N'*-(2,4-dihydroxybenzylidene)-4-hydroxy-2-oxo-1,2-dihydroquinoline-3-carbohydrazide**


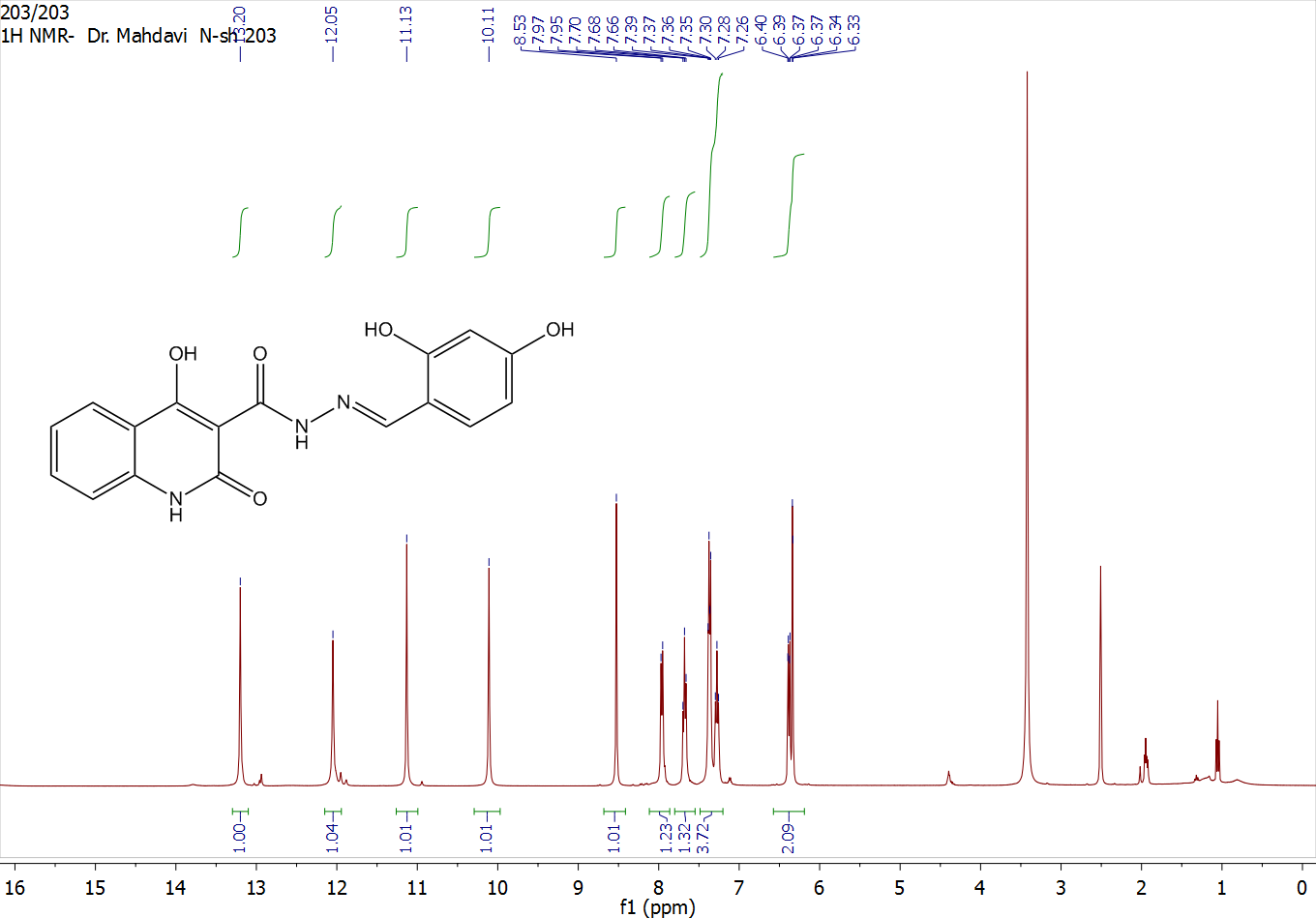


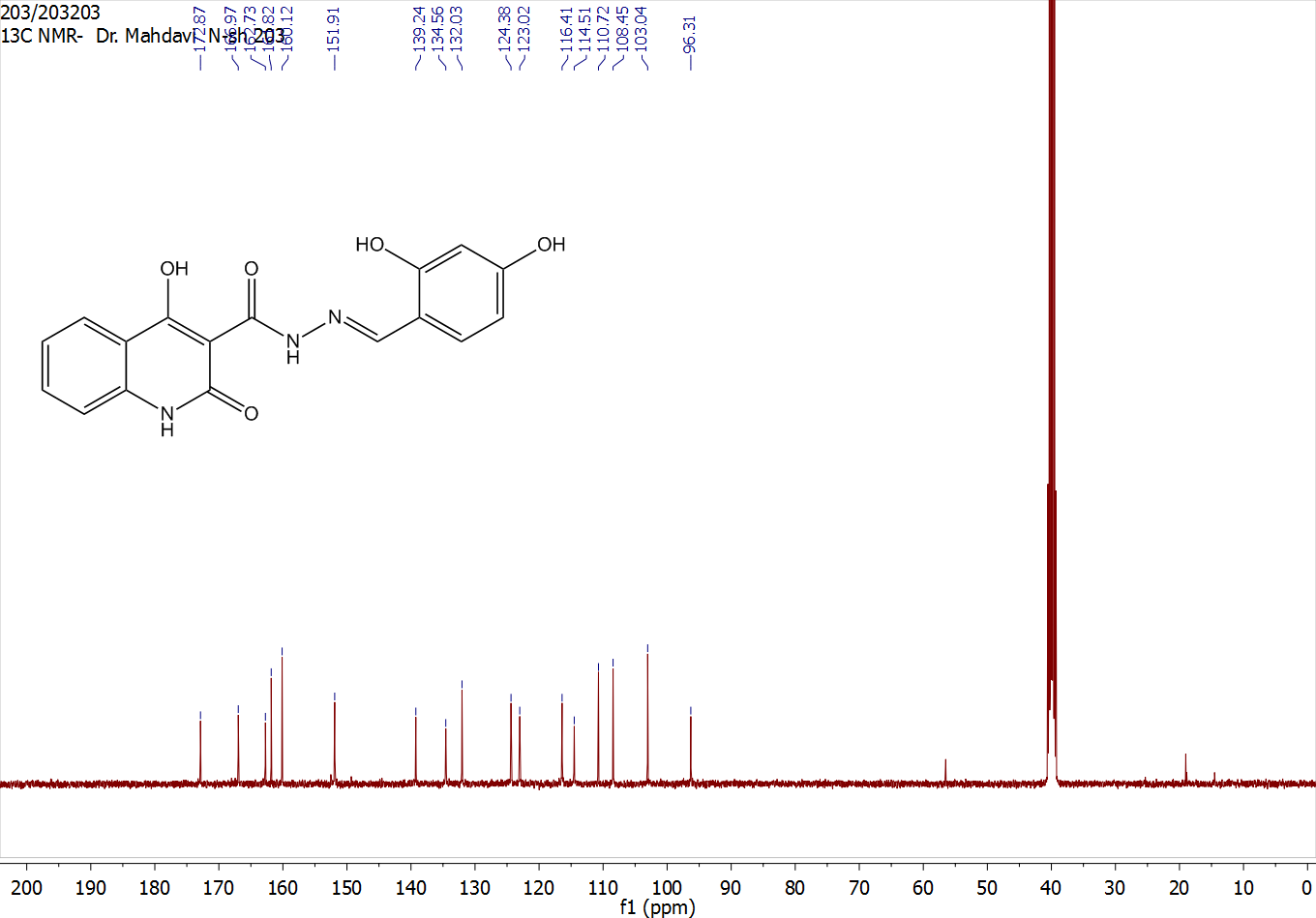


**(*E*)-4-hydroxy-*N'*-(4-hydroxy-3-methoxybenzylidene)-2-oxo-1,2-dihydroquinoline-3-carbohydrazide**

**
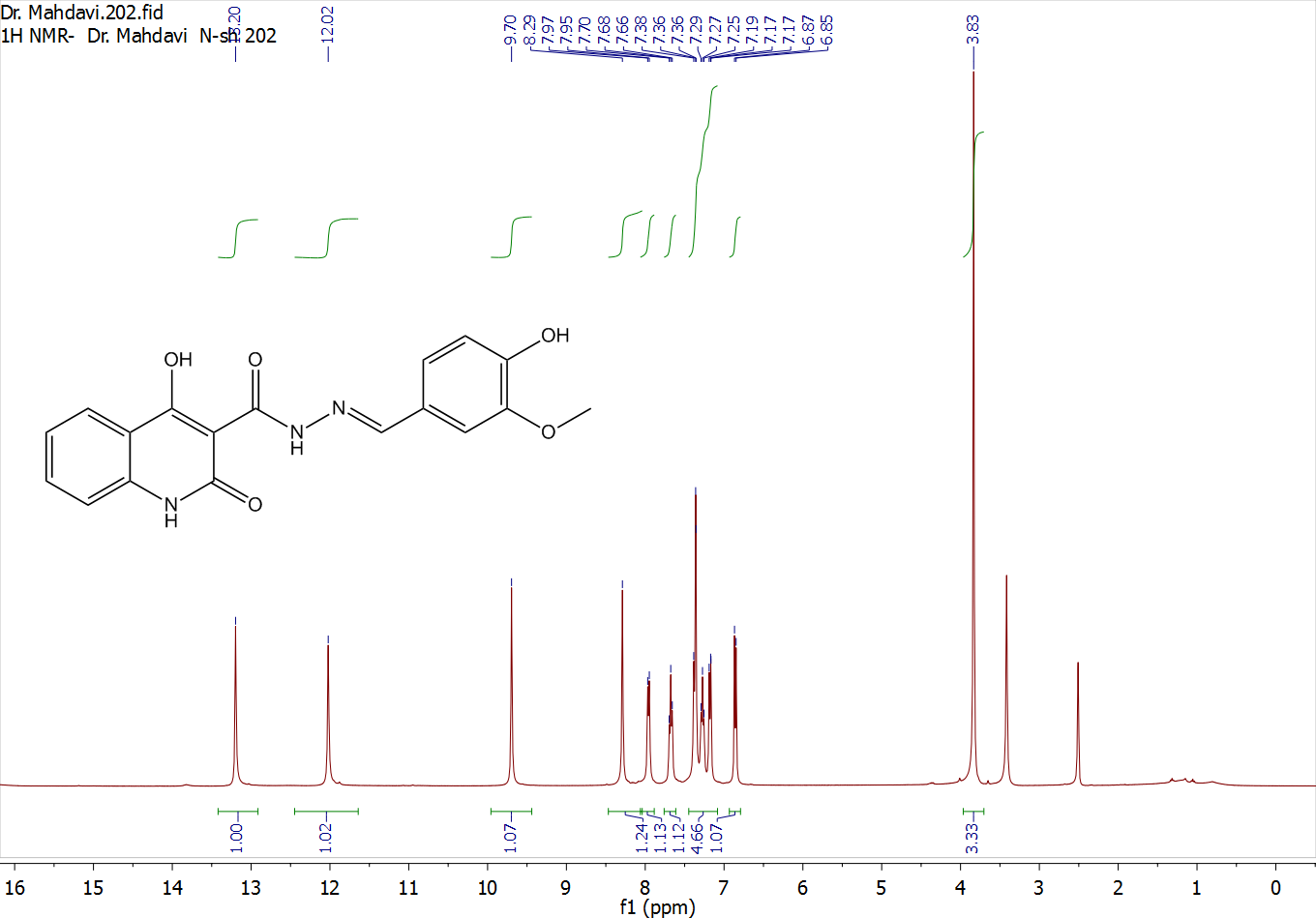
**

**
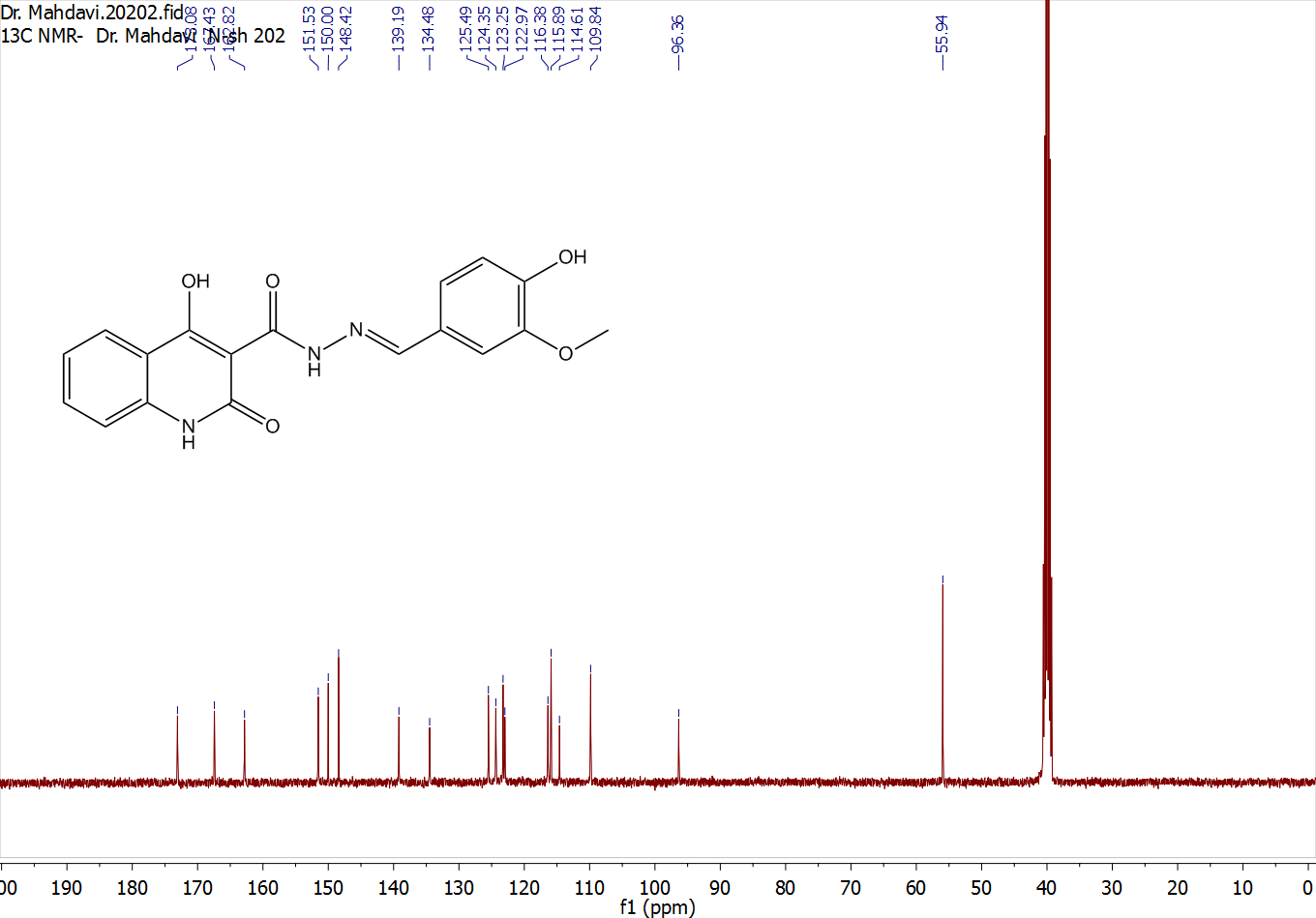
**
